# Strain-induced correlated metallic phase in 1T-TaS_2_

**DOI:** 10.1038/s41467-026-78883-1

**Published:** 2026-11-13

**Authors:** Shuto Suzuki, Kosuke Nakayama, Koki Yanagizawa, Katsuaki Sugawara, Seigo Souma, Kenichi Ozawa, Shunichi Takano, Ryoga Takahara, Takeshi Hara, Yusuke Wakabayashi, Takashi Takahashi, Tsutomu Nojima, Keiji Ueno, Takafumi Sato

**Affiliations:** 1 Department of Physics, Graduate School of Science, Tohoku University, Sendai, 980-8578 Japan; 2 Precursory Research for Embryonic Science and Technology (PRESTO), Japan Science and Technology Agency (JST), Tokyo, 102-0076 Japan; 3 International Quantum Materials Center (Q²), Advanced Institute for Materials Research (WPI-AIMR), Tohoku University, Sendai, 980-8577 Japan; 4 Center for Science and Innovation in Spintronics (CSIS), Tohoku University, Sendai, 980-8577 Japan; 5 Institute of Materials Structure Science, High Energy Accelerator Research Organization (KEK), Tsukuba, Ibaraki, 305-0801 Japan; 6 Institute for Materials Research (IMR), Tohoku University, Sendai, 980-8577 Japan; 7 Department of Chemistry, Saitama University, Saitama, 338-8570 Japan; 8 International Center for Synchrotron Radiation Innovation Smart (SRIS), Tohoku University, Sendai, 980-8577 Japan; 9 Mathematical Science Center for Co-creative Society (MathCCS), Tohoku University, Sendai, 980-8578 Japan

**Keywords:** Electronic properties and materials, Surfaces, interfaces and thin films

## Abstract

Layered transition-metal dichalcogenide 1T-TaS_2_ has long been considered a prime example of a Mott insulator driven by strong electron correlations. However, this view has been recently challenged by proposals which favor a band insulating state arising from interlayer dimerization. This ongoing debate opens a frontier in 1T-TaS_2_ research focusing on stacking order, and raises a fundamental question with broad implications for two-dimensional materials: can electronic phases be engineered by actively tuning interlayer coupling? Here, using micro-focused angle-resolved photoemission spectroscopy supported by first-principles calculations, we demonstrate that uniaxial strain drives 1T-TaS_2_ from its insulating charge-density-wave phase into a stable metallic phase. This metallic phase, distinct from hydrostatic-pressure-induced or non-equilibrium hidden phases, emerges via a strain-induced rearrangement in the interlayer stacking of the Star-of-David charge order, effectively disrupting interlayer dimerization. Furthermore, the emergent metallic phase exhibits a significant quasiparticle mass renormalization, a hallmark of strong electron correlations, indicating its correlated nature and proximity to a Mott instability. This finding necessitates a re-evaluation of the interplay between structural and strong correlations in 1T-TaS_2_. The present results establish uniaxial strain as a powerful method for tuning the interlayer coupling, opening pathways for exploring quantum phases inaccessible under ambient conditions.

## Abstract (web summary)

The nature of the insulating state in the layered transition-metal dichalcogenide 1T-TaS_2_ remains under debate. Here, the authors show that uniaxial strain alters the stacking of charge‑density‑wave layers, driving an insulator‑to‑metal transition and yielding a correlated metallic state.

## Introduction

**Fig. 1:**
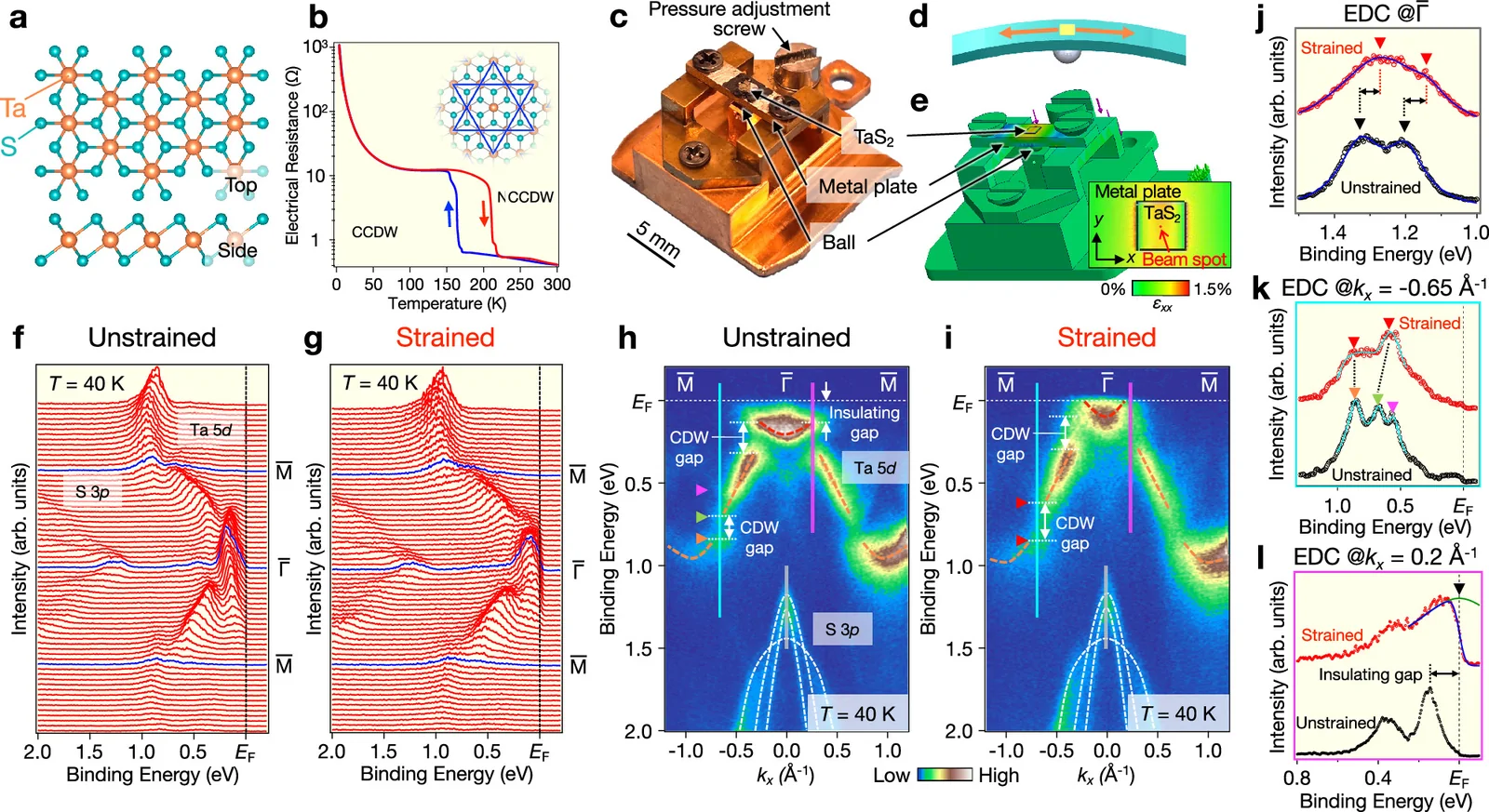
Change in the valence-band dispersion upon applying strain in 1T-TaS_2_. **a** Schematics of crystal structure of 1T-TaS_2_. **b** Temperature dependence of in-plane electrical resistivity upon heating and cooling. CCDW and NCCDW denote commensurate and nearly commensurate CDW, respectively. Inset shows a schematic of a star-of-David cluster. **c** Photograph of a uniaxial strain device. Sample is mounted on the metal plate and strained parallel to the a^*^ axis. In this device, a bulk TaS_2_ sample is mounted onto a metal plate with silver epoxy. By tightening the pressure-adjustment screw, the bridge and attached metal plate are mechanically bent downward against a metal ball, thereby generating tunable uniaxial strain on the sample. **d** Schematics of how the sample is uniaxially strained. **e** Distributions of *ε*_*xx*_ simulated using the finite element method. Inset shows an enlarged view around a 1 × 1 × 0.05 mm^3^ sample glued on CuBe plate. The beam spot size is shown by a red dot. **f**, **g** EDCs along the $\bar{\Gamma }\bar{{\mbox{M}}}$ cut for the unstrained and strained samples, respectively, measured at *T* = 40 K. **h**, **i** ARPES-intensity plot measured along the $\bar{\Gamma }\bar{{\mbox{M}}}$ cut for the unstrained and strained cases, respectively. Solid and dashed curves are a guide for the eyes to trace the band dispersion. **j** Comparison of EDCs at the $\bar{\Gamma }$ point between the unstrained and strained cases shown by grey vertical l**i**nes in (**h** and **i**), together with the numerical fittings (black curves). **k** Comparison of EDCs between the unstrained and strained cases at *k*_*x*_ = −0.65 Å^−1^ shown by cyan l**i**nes in (**h** and **i**), together with the results of numerical fittings (solid curves). **l** Comparison of EDCs between the unstrained and strained cases at **k**_F_ shown by magenta l**i**nes in (**h** and **i**). Blue curves are the fitting results for the strained case near *E*_F_, assuming Lorentzian curves (green curve) multiplied by the Fermi-Dirac distribution function.

The discovery and manipulation of quantum phases of matter is a central pursuit in condensed matter physics, promising fundamental insights and pathways to unexplored functionalities. A particularly exciting frontier in this quest involves the tuning of interlayer interactions in layered materials and heterostructures, exemplified by recent discoveries of unconventional superconductivity and correlated insulating states in twisted bilayer systems^1–3^. Among naturally occurring layered materials, 1T-TaS_2_ (Fig. 1a) stands out as an exceptional platform for exploring the crucial influence of interlayer effects. Its rich phase diagram includes multiple charge-density-wave (CDW) phases that vary with temperature^4–8^. Particularly important is the insulating commensurate CDW (CCDW) phase observed below ~150 K (Fig. 1b). The key framework is an intralayer √13 × √13 triangular superlattice of the Star-of-David (SoD) clusters^4,5,9,10^. Each SoD cluster consists of thirteen Ta atoms, with twelve contracting toward a central one (Fig. 1b, inset), leaving one unpaired electron on the central Ta atom. Simple band theory predicts this configuration should yield a half-filled band and thus metallic behaviour^11^, starkly contrasting the observed insulating nature. To resolve this discrepancy, Fazekas and Tosatti proposed in 1979 that strong electron correlations drive a Mott transition, localizing the unpaired electron on each SoD cluster^5,9^. The absence of magnetic ordering down to the lowest temperatures, combined with the triangular arrangement of these localized electrons, further makes 1T-TaS_2_ a candidate material for a quantum spin liquid state^12–15^. These considerations suggest that the CCDW phase of 1T-TaS_2_ is a manifestation of an intricate interplay between reduced dimensionality and strong correlations.

However, a significant challenge to this Mott scenario emerged more recently. Studies proposed that interlayer dimerization, involving the pairing of electrons at the SoD centers in adjacent layers (specifically, the AL stacking configuration), results in a band insulator with a doubled unit cell along the *c* axis^16,17^. While earlier experiments supported the Mott insulator picture^4–6,18–20^, the prevailing view has increasingly shifted towards a band insulator description driven by interlayer coupling^16,17,21^, highlighting that interlayer interactions are pivotal in determining the ground state properties of 1T-TaS_2_. This debate is linked to fundamental questions regarding the competition between intralayer Mott physics and interlayer coupling/hopping effects in layered quantum materials^22,23^.

These developments have created a research focus on the interlayer stacking degree of freedom within the SoD lattice. Theoretical work suggests that both ground state energy and electronic properties are sensitive to specific stacking patterns (e.g., AL vs L stacking, corresponding to with or without interlayer dimerization, respectively), predicting potential transitions between Mott, band insulating, or metallic states^16,17,21^. Experimental evidence is consistent with this prediction, supporting a transition from a band insulator to a Mott insulator when interlayer dimerization is disrupted, for instance, through surface termination effects and stacking faults^24–26^. Given the sensitivity to stacking, a crucial question arises: can the interlayer coupling of the SoD lattice be actively and controllably tuned to explore and stabilize phases beyond those currently observed? While hints of hidden metallic states coexisting with SoD lattices have been reported, such as on localized surface regions^24,27^ or under non-equilibrium stimuli (e.g., electric or light pulses)^28–31^, their deliberate creation and control as stable phases under equilibrium conditions have remained elusive^32^. Furthermore, the mechanisms driving such insulator-to-metal transition and the nature of emergent metallic phases are still largely unclear, partly due to the difficulty in directly visualizing the electronic band structure across the transition.

To address the challenge of controlling structural and electronic properties under equilibrium conditions, strain engineering, particularly uniaxial strain, offers a powerful approach^33–41^. Unlike hydrostatic pressure, which has enabled breakthroughs often under extreme conditions (e.g., the synthesis of near-room-temperature superconductors^42,43^ and the enhancement of transition temperatures in various superconductors^44–46^), uniaxial strain could provide more targeted control of electronic phases even at modest strain levels ( ≤ 1%), by selectively altering specific structural degrees of freedom and anisotropies^38–40,47,48^. Notably, recent advancements integrating angle-resolved photoemission spectroscopy (ARPES) with uniaxial-strain devices have revealed intriguing phenomena in layered materials. Examples include Lifshitz transitions^49^, suppression of Mott insulating states^50^, topological phase transitions^51,52^, and modulations of CDW gaps^53,54^. These advances highlight the combined ARPES and uniaxial strain approach as a uniquely suited tool for manipulating and directly probing electronic properties in layered materials under equilibrium conditions.

In this work, we apply micro-focused ARPES coupled with a custom-designed uniaxial-strain device to investigate the response of 1T-TaS_2_. Our results demonstrate a clear insulator-to-metal transition under uniaxial strain. Comparison of our ARPES data with density functional theory (DFT) calculations for different interlayer stacking sequences^17^ suggests a strain-induced π-phase shift of the interlayer SoD stacking as the mechanism responsible for metallicity. This contrasts sharply with non-equilibrium phenomena^28–31^ and high-pressure-induced transitions^55,56^ which modify intralayer SoD lattice structure. Importantly, our work further reveals strong correlation effects in this strain-induced metallic state. This suggests that while interlayer dimerization may dominate in the ground state of pristine 1T-TaS_2_, underlying strong correlation physics remains crucial and becomes electronically manifest once the dimerization gap closes via structural tuning. This work opens avenues for exploring and manipulating correlated phenomena through the active control of interlayer coupling.

## Results

### Change in the valence-band dispersion under strain

To investigate the strain-induced modifications of the electronic structure in 1T-TaS_2_, we developed a compact uniaxial strain device compatible with ARPES measurements (Fig. 1c). The device applies tensile strain along a specific crystallographic direction, i.e., //a^*^ axis in the present study, as illustrated in Fig. 1d. Finite-element simulations (Fig. 1e) and strain-gauge measurements (Supplementary Note 1) confirmed that our setup achieves controlled uniaxial strain on the order of 1%. As an independent estimation, we performed an ex situ laser microscopy on a representative TaS_2_ crystal mounted in the strain device. This analysis reveals a local in-plane strain *ε*_*xx*_ ≈ 1.5% at the centre of the sample, in reasonable agreement with the finite-element simulations (Supplementary Note 1). While the simulated strain macroscopically varies from ~1.3% at the centre of the sample to ~0.2% near the edges, within the ~12-μm width of the beam spot in our micro-ARPES system^57^ (see red dot), this gradient corresponds to a strain variation of only ~0.03%, which is two orders of magnitude smaller than the applied maximum strain. Therefore, the use of micro-focused ARPES allows us to probe regions with relatively uniform strain.

We first characterized the electronic structure of 1T-TaS_2_ in the absence of uniaxial strain. Figure 1f presents energy distribution curves (EDCs) along the $\bar{\Gamma }\bar{{\mbox{M}}}$ cut of the surface Brillouin zone, acquired at *T* = 40 K in the CCDW phase. EDCs under strain are also shown in Fig. 1g. One can clearly recognize several highly dispersive bands in both EDCs, confirming good crystallinity of our sample. The corresponding ARPES intensity plot for the unstrained sample in Fig. 1h reveals hole-like bands at high binding energy (*E*_B_) near $\bar{\Gamma }$, which originate from the S 3*p* orbitals (white dashed curves). Within ~1 eV of the Fermi level (*E*_F_), multiple bands derived from the Ta 5 *d* orbitals are observed, exhibiting characteristic features of the (√13×√13)R13.9° CDW superlattice potential. Specifically, band folding and hybridization give rise to discontinuities and suppressed spectral intensity within *E*_B_ ranges of 0.15–0.35 eV and 0.7–0.9 eV, corresponding to partial CDW gaps. An insulating gap of ~0.15 eV also opens at *E*_F_. These features are consistent with previous ARPES reports in the CCDW phase of 1T-TaS_2_ (refs. ^16,58–60^).

Next, a comparison of the band structure before and after applying strain (Fig. 1f, h and 1g, i, respectively) shows several key features. First, the S 3*p* bands shift upward in energy (by ~0.06 eV) under strain, as evident in the EDCs at the $\bar{\Gamma }$ point (Fig. 1j, indicated by red and black triangles). Second, the CDW gap magnitude appears to increase (Fig. 1k), as the energy separation of the gap edges is larger. This suggests the persistence of the primary CCDW structure. Third, most notably, the insulating gap at *E*_F_ closes completely under strain. This is clearly seen in the EDCs at the Fermi wave vector (**k**_F_) points in Fig. 1l, where spectral weight suppression near *E*_F_ is lifted, and a metallic Fermi edge emerges. Fitting the leading edge with a Lorentzian peak centred at *E*_F_ (green curve), multiplied by the Fermi-Dirac distribution (black curve), reproduces the experimental data well. This provides direct spectroscopic evidence for a strain-induced insulator-to-metal transition in 1T-TaS_2_ under equilibrium conditions (see Supplementary Note 2 for the robustness of the gap closing). Importantly, this transition is fully reversible upon releasing the applied strain (Supplementary Note 3). This demonstrates that the uniaxial strain induces an elastic deformation without causing irreversible structural damage.

### Influence of strain on the Fermi surface and chiral pattern

**Fig. 2:**
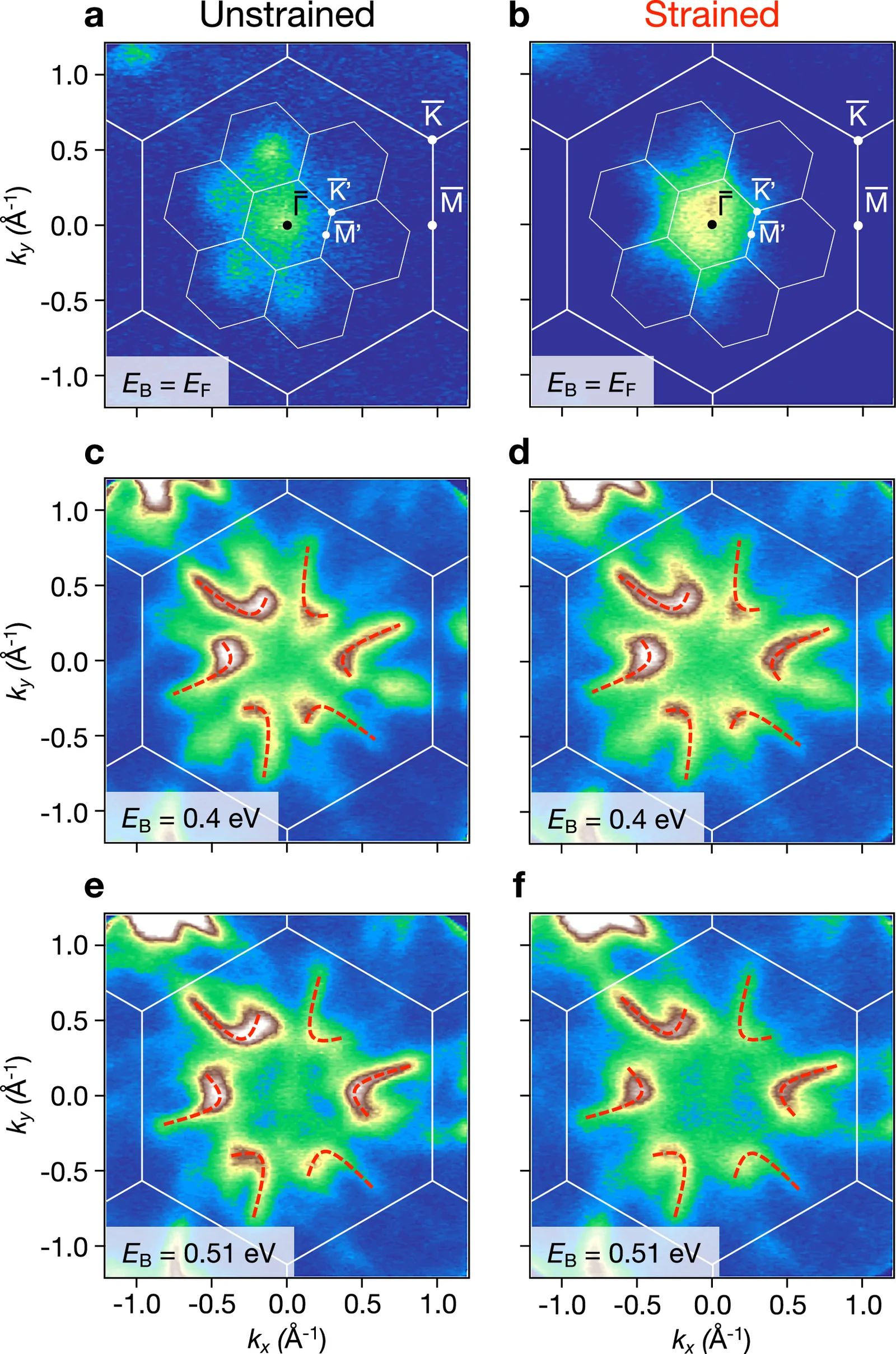
Chiral pattern in the energy contours. **a,** **b** ARPES-intensity map at *E*_F_ plotted as a function of two-dimensional wave vector, *k*_*x*_ and *k*_*y*_, for the unstrained and strained cases, respectively, measured at *T* = 40 K. **c,** **d** Same as **a** and **b**, but at *E*_B_ = 0.4 eV. Red dashed curves are a guide for the eyes to trace the chiral pattern. **e,** **f** Same as **c** and **d**, but at *E*_B_ = 0.51 eV.

To visualize the consequences of the gap closure at *E*_F_, we performed ARPES-intensity mapping as a function of in-plane momentum (*k*_*x*_, *k*_*y*_). Figure 2a, b compares the constant-energy contours at *E*_B_ = *E*_F_ before and after applying strain. In the unstrained insulating phase (Fig. 2a), only a weak residual spectral weight appears around the $\bar{\Gamma }$ point, modulated with the periodicity of (√13×√13)R13.9° reconstructed Brillouin zone (thin hexagons), reflecting the tail of the valence band below *E*_F_. Under strain (Fig. 2b), on the other hand, a pronounced circular Fermi pocket emerges at $\bar{\Gamma }$, indicative of the metallic state. Importantly, this Fermi surface is distinct from that of the high-temperature ( > 350 K) normal metallic phase (i.e., incommensurate CDW, ICCDW, phase), which is characterized by the elongated electron pockets at the $\bar{{\mbox{M}}}$ points^61,62^. Thus, although both phases are metallic, the strain-induced state is a fundamentally different electronic phase.

Further insight into the underlying structure of the strain-induced metallic phase is obtained from the constant-energy map at *E*_B_ = 0.4 eV close to the CDW-gap edge, where CDW effects are more prominent (Fig. 2c, d). In both strained and unstrained samples, a windmill-like intensity pattern is observed. This pattern breaks the symmetry of the original Brillouin zone (thick white lines) and has been attributed to the selective observation of specific CCDW domains (specifically, clockwise and counterclockwise rotations of the (√13×√13)R13.9° superlattice) in micro-ARPES measurements^16,63^; in our case, the clockwise domain is selectively probed. This pattern is also observed at deeper *E*_B_’s, e.g., at 0.51 eV (Fig. 2e, f). The persistence of this chiral pattern under strain strongly suggests that the fundamental in-plane √13×√13 SoD cluster arrangement within the layers remains intact despite the insulator-to-metal transition. This is further corroborated by the observation of core-level splitting and intensity modulation following the periodicity of the (√13×√13)R13.9° potential, as detailed in Supplementary Note 4. These results point toward the strain-induced emergence of a metallic state coexisting with the commensurate SoD lattice.

### Comparison with DFT and the origin of the metallic state

**Fig. 3:**
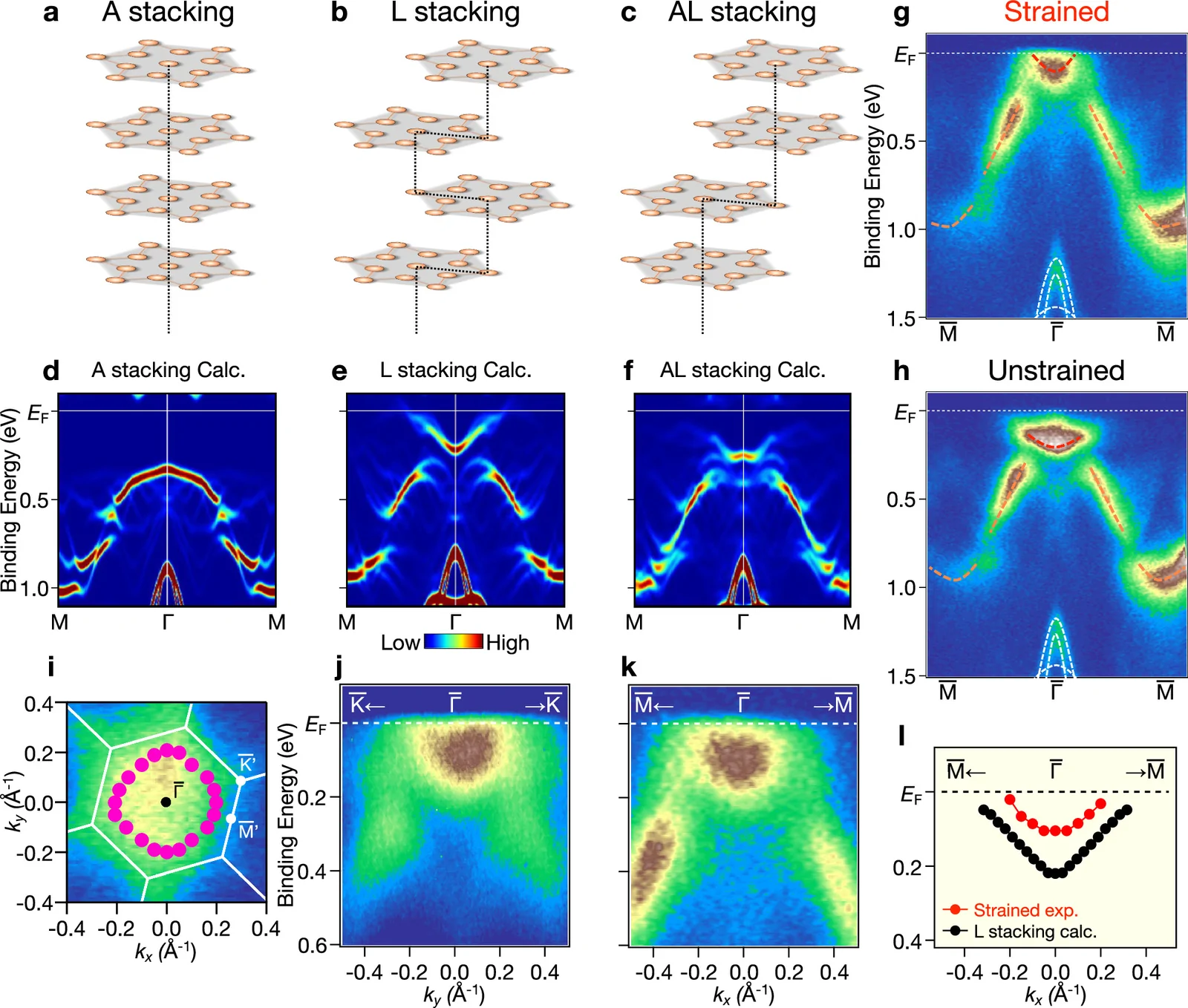
Comparison with DFT calculations and the origin of strained metallic phase. **a**–**c** Schematics of A, L, and AL stacking configurations, respectively. **d**–**f** Calculated electronic structures for A, L, and AL stacking configurations respectively, along the $\bar{\Gamma }\bar{{\mbox{M}}}$ line, adapted with permission from ref. ^17^. Copyrighted by the American Physical Society. **g,** **h** ARPES intensity of 1T-TaS_2_ for the strained and unstrained cases, respectively, reproduced from Fig. 1i, h. **i** ARPES-intensity map for the strained case at *E*_F_ in the folded Brillouin zone. Magenta dots are extracted from peak positions of second-derivative ARPES intensity. **j,** **k** ARPES-intensity plot for the strained case, measured along the $\bar{\Gamma }\bar{{\mbox{K}}}$ and $\bar{\Gamma }\bar{{\mbox{M}}}$ cuts, respectively. **l** Comparison of the band dispersions between the calculation for the L stacking configuration (black dots) and the experimental results (red dots).

Since the in-plane commensurate SoD lattice is preserved, we now discuss the origin of the intriguing metallic phase by considering the interlayer stacking sequence of the SoD lattice. As illustrated in Fig. 3a–c, three representative stacking configurations have been identified^17^. In the A stacking, the centre Ta atoms of each SoD cluster align vertically along the *c* axis. In the L stacking (Fig. 3b), however, SoD clusters are shifted laterally such that the Ta atoms at the vertices of SoD cluster in one layer vertically connect to the centre Ta atoms of the adjacent layer, introducing a π-phase shift relative to the A stacking. The AL stacking (Fig. 3c) is a bilayer structure where pairs of A-stacked layers are stacked with an L-type shift, producing dimerized pairs of SoD-lattice layers along the *c* axis. Density-functional-theory (DFT) calculations^17^ indicate that the band structure in the CCDW phase is strongly affected by the stacking pattern. Along the $\bar{\Gamma }\bar{{\rm{M}}}$ cut of the bulk Brillouin zone, no states appear within 0.3 eV of *E*_F_ for the A stacking (Fig. 3d), whereas an electron band crosses *E*_F_ in the L stacking (Fig. 3e). For the AL stacking, an electron-like band is visible, but does not cross *E*_F_, and a gap opens along this cut (Fig. 3f) due to the interlayer dimerization inherent to this configuration.

Comparing these calculated band structures with ARPES data (Fig. 3g, h) provides crucial insights. In the unstrained phase (Fig. 3h), the measured dispersion closely matches the AL stacking calculation, particularly regarding the topmost valence-band position at $\bar{\Gamma }$ and the presence of a band gap. This supports the picture of interlayer dimerization driving the insulating state in pristine bulk 1T-TaS_2_. Under uniaxial strain (Fig. 3g), however, the observed electron band crossing *E*_F_ is well reproduced by the L stacking calculation, rather than by the AL stacking (note that some band splitting, e.g., those predicted in the energy range of 0.3-0.4 eV, are not well resolved, but their invisibility does not necessarily contradict the L-stacking scenario; instead, it is most likely due to the weak spectral intensity and experimental spectral broadening, as supported by an analogous situation in the unstrained pristine phase). These results strongly suggest that the applied strain induces a rearrangement in the interlayer stacking from the dimerized AL configuration to the L-like configuration, while maintaining the in-plane commensurability of the SoD lattice. Such a rearrangement is plausible, as theoretical studies suggest the AL and L stackings are close in energy^17^. Furthermore, the in-plane tensile strain can induce the out-of-plane compression via the Poisson effect, potentially making the L stacking, which avoids direct vertical alignment of the central Ta atoms of the SoD clusters, energetically favorable due to a reduction in Coulomb repulsion at decreased interlayer spacing. Indeed, the representative *ε*_*xx*_ value ≈ 1.5% gives an out-of-plane strain of *ε*_*zz*_ = −*νε*_*xx*_ ≈ −0.4% (Supplementary Note 1) that is comparable to the 0.3% compression at which prior calculations place the AL and L stackings near degeneracy^17,32^. It is remarked that the stacking transition could be primarily localized at the surface because interlayer slip effect is expected to be most pronounced at the surface due to the macroscopic shear nature of the applied strain^64,65^ in the present experimental setup (Fig. 1c–e; see also Supplementary Note 5), consistent with x-ray diffraction measurements (Supplementary Note 6). Based on the surface sensitivity of ARPES and a simple escape-depth estimation, a reconstructed region is suggested to include the topmost two TaS_2_ layers (Supplementary Note 6). Such a near-surface L-like reconstruction may involve partial interlayer slip and/or local stacking faults that weaken the AL-type interlayer dimerization (Supplementary Note 5). Alternative mechanisms, including a mere destruction of the SoD cluster or a transition to the metallic nearly commensurate CDW (NCCDW in Fig. 1b) phase, are inconsistent with our observations of the persistent chiral pattern (Fig. 2d, f) and the different band structure compared to the NCCDW phase (see Supplementary Note 7). A uniform change in the lattice constants would also not, by itself, close the gap as long as the AL-type interlayer dimerization persists. It is noted that, while the existence of three symmetry-equivalent L sites leads to stacking disorder in the absence of strain^16,31^, it is plausible that uniaxial strain breaks the three-fold in-plane rotational symmetry of the lattice, thereby lifting the degeneracy of the three L sites. The uniaxial strain would thus selectively suppress the random orientations and promote macroscopic long-range stacking order.

Our findings are distinct from previous investigations into tuning 1T-TaS_2_. Previous demonstrations of metallic states typically relied on non-equilibrium stimuli, such as current, voltage, or light pulses^28–31^, which are often transient effects and not only tune interlayer interactions but presumably result in ICCDW- or NCCDW-like in-plane lattices due to thermal excitations and carrier injection inherent to these routes^28,31^. In stark contrast, we achieve a stable metallic state under equilibrium conditions using uniaxial strain. Furthermore, the observed transition involves interlayer sliding between adjacent layers while crucially preserving the in-plane commensurate SoD lattice. It is noted that while the previous study applying uniaxial strain of ~0.2 % reported a decrease in the band/Mott gap size, the system remains gapped^32^. In stark contrast, our work provides a clear visualization of a genuine insulator-to-metal transition. In addition, the stabilization of the metallic phase enables us to analyze the quasiparticle dispersion and evaluate correlation effects as detailed later, whereas such information could not be obtained in the literature because the system remained gapped. Moreover, the emergent metallic state in this work differs from that induced by hydrostatic pressure, which triggers a transition to the NCCDW phase^55,56^. Therefore, our work establishes equilibrium uniaxial strain as a selective tool to induce a unique metallic phase in 1T-TaS_2_ by primarily controlling the stacking degree of freedom, distinct from previously induced states.

### Correlated nature of the strain-induced metallic phase

Having established the strain-induced transition to a metallic phase, we next examine its spectral characteristics to understand its electronic nature. By tracing the second derivative of the ARPES intensity, we determined **k**_F_’s of the electron pocket (Fig. 3i). Assuming a circular shape for the pocket, we estimate the Fermi-surface area of *S*_FS_ ~ 0.13 Å^-2^. This area corresponds to approximately 50% (52%) of the reconstructed Brillouin zone (white hexagons), consistent with the expected half-filling of the band derived from the single unpaired electron per SoD in the commensurate lattice without interlayer dimerization, again confirming our interpretation. To reveal the electron-band dispersion, we analyzed the ARPES intensity near *E*_F_ along the $\bar{\Gamma }\bar{{\mbox{K}}}$ and $\bar{\Gamma }\bar{{\mbox{M}}}$ cuts (Fig. 3j, k, respectively), and found a band bottom at 0.11 eV below *E*_F_ (red circles in Fig. 3l). In contrast, DFT calculations for the L stacking predict a band bottom at a much larger *E*_B_ of 0.22 eV (black circles). This substantial discrepancy highlights a strong renormalization of the electron band, with an effective mass renormalization factor of ~2 (0.22/0.11), providing evidence that the strain-induced metallic phase in 1T-TaS_2_ is not a simple metal but a strongly correlated metallic state characterized by heavy quasiparticles.

This correlated character further distinguishes the observed metallic state from the weakly correlated high-temperature metallic phases of 1T-TaS_2_. While strong electron correlations inherent to the commensurate SoD lattice are expected, they are largely masked in pristine 1T-TaS_2_ due to the opening of a band gap associated with interlayer dimerization. On the other hand, uniaxial strain closes this gap, thereby unveiling and bringing to the forefront the profound influence of these intrinsic strong correlations on the quasiparticle bands near *E*_F_. The observed mass renormalization factor of approximately 2 is as large as the value found in archetypal correlated metals such as SrVO_3_ (refs. ^66,67^), which is known to reside in proximity to a Mott transition^68,69^. The similarity suggests that the strain-induced metallic phase in 1T-TaS_2_ is located at the verge of a Mott transition. This finding underscores the critical and intertwined roles of interlayer stacking and many-body effects in shaping the electronic properties of 1T-TaS_2_, effectively bridging the previously proposed, distinctly different pictures, band insulator vs Mott insulator.

Our demonstration of the transition from an insulator to a correlated metal, driven by a strain-induced manipulation of interlayer stacking of commensurate SoD lattices, carries broad implications. From a fundamental perspective, the principle of stacking engineering via strain established here offers a powerful pathway to explore and stabilize electronic, magnetic, or topological phases in other transition-metal dichalcogenides and layered compounds, where interlayer interactions are pivotal. Furthermore, the strain-induced correlated metal state itself presents a fertile ground for explorations of strong correlation physics. Its proximity to a Mott transition makes it an ideal platform for investigating emergent phenomena such as quantum criticality, non-Fermi liquid behaviour, and potential instabilities towards exotic ordered states including unconventional superconductivity, which are often found on the verge of an insulator-to-metal transition^70^. From an applied perspective, our results open a viable route toward designing straintronic devices. The ability to induce a dramatic phase change (insulator-to-metal) with modest equilibrium mechanical deformation suggests a potential for high-performance sensors, switches, and memory elements. This mechanical control could potentially be integrated into other external stimuli (e.g., electrostatic gating, optical excitation) to realize multi-functional devices with enhanced tunability.

## Methods

### Sample growth and characterization

High-quality single crystals of 1T-TaS_2_ were grown by the chemical vapour transport method. The in-plane crystallographic orientation was determined with Laue x-ray back scattering measurements. Electrical resistivity measurements were performed using an Oxford Instruments cryostat with 8 T superconducting magnet at Center for Low Temperature Science in Tohoku University. Measurements were performed over a temperature range from 2 K to 300 K, with the temperature change rate set between 0.5 K and 1 K per minute.

### Uniaxial strain device

A bulk TaS_2_ crystal with typical dimensions of 1 × 1 × 0.05 mm^3^ was mounted with silver epoxy onto a CuBe plate. By tightening the pressure-adjustment screw, the bridge and the attached metal plate are mechanically bent downward against a metal ball, thereby applying tensile strain to the crystal along the a* axis (Fig. 1c, d). The magnitude of the applied strain was calibrated in three independent ways, described in the following three subsections.

### Strain calibration using strain gauges

The strain applied to the sample plate was measured at room temperature using commercial strain gauges (KYOWA KFRS-02-120-C1-13). The gauge reading increases with the rotation angle of the pressure-adjustment screw and reaches 0.5% at maximum. Because the 0.8-mm width of the gauge averages over the macroscopic strain gradient along *x*, this value is smaller than the local strain at the centre of the sample (Supplementary Note 1).

### Strain calibration using laser microscopy

Laser-microscope images of a TaS_2_ crystal mounted on the same strain device were acquired at room temperature in air using a KEYENCE VK-X1100 laser scanning confocal microscope, before and after applying uniaxial strain along the *x-*axis. Line profiles were extracted near the centre of the crystal, at a position geometrically corresponding to the region probed by micro-ARPES, and the local axial strain along *x* (*ε*_*xx*_) was evaluated from the change in the lateral distance between matched surface features (Supplementary Note 1).

### Finite-element simulations

The finite-element simulations were performed using SOLIDWORKS Simulation. For the metal plate made of CuBe, a Young’s modulus of 115 GPa and a Poisson’s ratio of 0.3 were implemented. The inset in Fig. 1e shows the simulated spatial distribution of *ε*_*xx*_ on the TaS_2_ sample with realistic dimensions of 1 × 1 × 0.05 mm^3^ glued on a CuBe plate. The absence of a strain gradient along the *y-*axis supports the uniaxial nature of the applied strain.

### ARPES measurements

ARPES measurements were performed using a Scienta-Omicron DA30 spectrometer at BL-28A in Photon Factory, KEK, with a micro-focused beam spot of 10 × 12 µm^2^ (ref. ^57^). Circularly polarized photons with photon energy of *hν* = 98 eV were used to probe the *k*_*z*_ = 0 plane. Energy and angular resolutions were set to be 35 meV and 0.3°, respectively. The base pressure during the measurements was better than 1.0 × 10^−10^ Torr. The Fermi level was referenced to that of a polycrystalline gold film. Uniaxial strain was applied in two paths. In path 1, samples were strained ex situ, then installed in UHV, cooled down to 40 K, cleaved in situ, and measured. In path 2, samples were installed in UHV, first cooled to 40 K, cleaved in situ, and measured without strain. Afterwards, the samples were strained in situ and measured again. In both paths, similar results were obtained under uniaxial strain (see Supplementary Note 8). In the main text, the results obtained for path 2 were presented to compare the electronic states with and without strain.

### X-ray diffraction measurements

X-ray diffraction measurements under uniaxial strain were performed using the same strain device, which was attached to a closed-cycle refrigerator. The experiments were conducted using a Rigaku 1.2-kW rotating anode x-ray generator with a VariMax-HF multilayer monochromator. The diffracted x-rays were collected by a two-dimensional XPAD S70 photon-counting pixel detector, which has an active area of 15 mm × 75 mm and a pixel size of 130 μm × 130 μm. Reciprocal-space maps parallel to the *h*0*l* plane were obtained at *T* = 50 K, well below the CDW transition temperature of pristine TaS_2_ (Supplementary Note 6).

### First-principles calculations

First-principles calculations in Fig. 3d–f were based on the density functional theory (DFT) within the generalized gradient approximation (GGA)^17^. The cut-off energy was set at 260 eV, and the *k*-point mesh was 4 × 4 × 8 for the Brillouin zone of the √13×√13×1 cell. All atoms were relaxed, and the calculated equilibrium lattice constants (*a* = 3.35 Å and *c* = 5.79 Å) reasonably agree with the experimental values (*a* = 3.36 Å and *c* = 5.92 Å).

## Supplementary information

Supplementary Information

Peer Review file

## Data Availability

The ARPES data generated in this study are available within the main text and Supplementary Information. The corresponding raw ARPES data have been deposited in the Zenodo database under accession code 10.5281/zenodo.22810375 (https://doi.org/10.5281/zenodo.22810375). The transport data are provided in Fig. 1b. The x-ray diffraction and laser-microscopy data are provided in the Supplementary Information.
