# Supplementary material for: Strain-induced correlated metallic phase in 1T-TaS2
Source: Nat Commun. 2026 Nov 13;17:11407. doi: 10.1038/s41467-026-78883-1 (PMC13768348; doi:10.1038/s41467-026-78883-1)
Supplement: Supplementary file 1 [file 41467_2026_78883_MOESM1_ESM.pdf]

Supplementary information for "**Strain-induced correlated metallic phase in 1T-TaS_2**", by Shuto Suzuki *et al*.

Supplementary Note 1: Evaluation of strain

The actual magnitude of the applied strain was evaluated by using commercial strain gauges (KYOWA KFRS-02-120-C1-13) at room temperature (see Supplementary Fig. 1a for the measurement configuration). As shown in Supplementary Fig. 1b, upon rotating the screw of the device, the strain measured by the gauge increases, reaching a maximum of 0.5%. The smaller magnitude of the measured strain than the simulated value (1.3%) is attributed to the spatial averaging effect; as mentioned in the main text, there is a macroscopic gradient of the strain along x, and the dimension of the strain gauge used in this study is 0.8 mm in width, so that the measured results represent a spatial average of the strain distribution. Therefore, the 0.5% sets the minimum strain magnitude and the actual maximum strain must be larger.

To further quantify the strain at the sample surface, we measured the local in-plane strain ε_{xx} using laser microscopy. Because the TaS_2 sample used here was different from the one used for ARPES but was mounted on the same strain device (Supplementary Fig. 2a), the resulting values should be regarded as representative estimates of the strain scale relevant to the ARPES

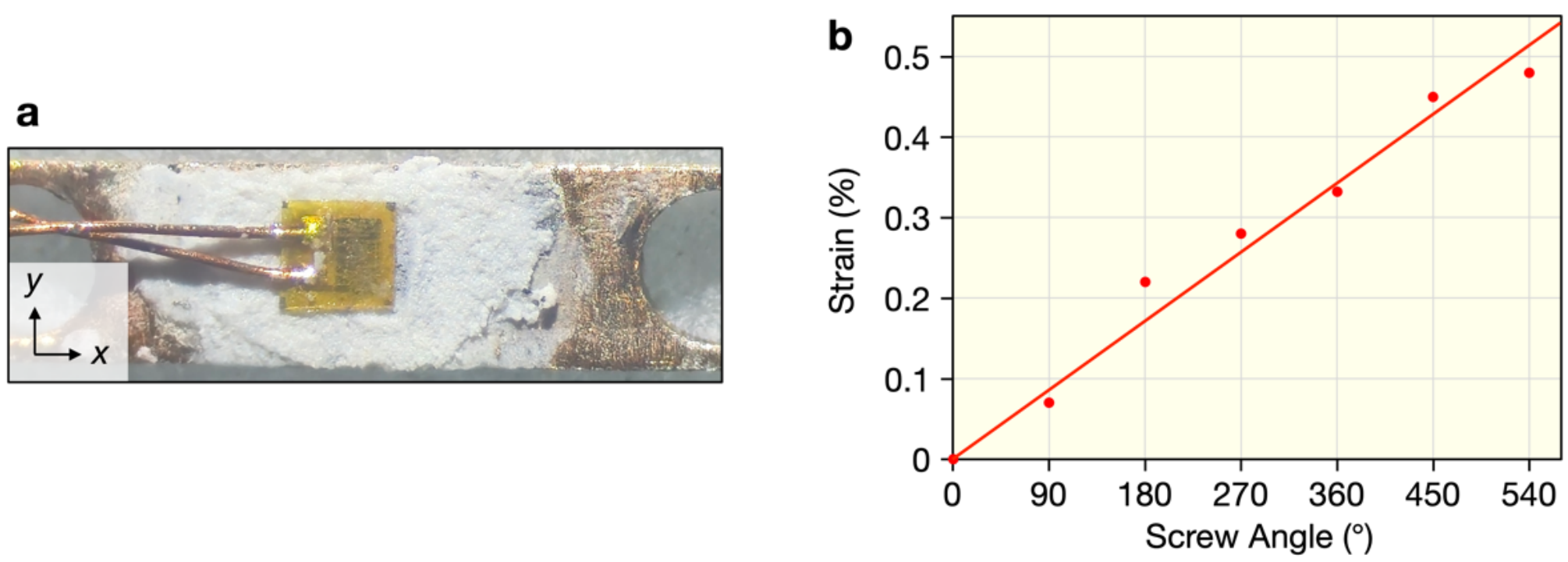

Supplementary Fig. 1 | Characterization of applied strain. **a** Optical microscope image of the strain gauge on the sample plate. **b** Plot of strain gauge readings against rotation angle of the pressure adjustment screw (red dots) with a linear fit (red line).

observation. We acquired laser-microscope images of the TaS_2 crystal at room temperature in air using a KEYENCE VK-X1100 laser scanning confocal microscope before and after applying uniaxial strain along the x axis (Supplementary Fig. 2b,c, respectively). Bright and dark features associated with surface morphology were visible in both images and served as spatial markers. We extracted line profiles along the red line near the centre of the crystal, at a position geometrically corresponding to the region nominally probed by micro-ARPES, and observed an increase in the lateral distance between matched features (compare the black and red dotted lines in Supplementary Fig. 2d). This analysis reveals a local in-plane tensile strain $\varepsilon_{xx} \approx 1.5\%$, in reasonable agreement with the ~1.3% value obtained from our finite-element simulations. We therefore used 1.5% for the following estimate of ε_{zz}.

The measured ε_{xx} allows us to estimate the resulting out-of-plane strain ε_{zz}. Using a Poisson ratio of $\nu = 0.26$ previously adopted for TaS_2 (ref. 1), the representative ε_{xx} value ≈1.5% gives $\varepsilon_{zz} = -\nu\, \varepsilon_{xx} \approx -0.39\%$. This value is comparable to the 0.3% reduction in interlayer spacing at which prior calculations place the AL and L stackings near degeneracy[2,3]. The in-plane tension and resulting out-of-plane compression may therefore reduce the energy cost of an L-like

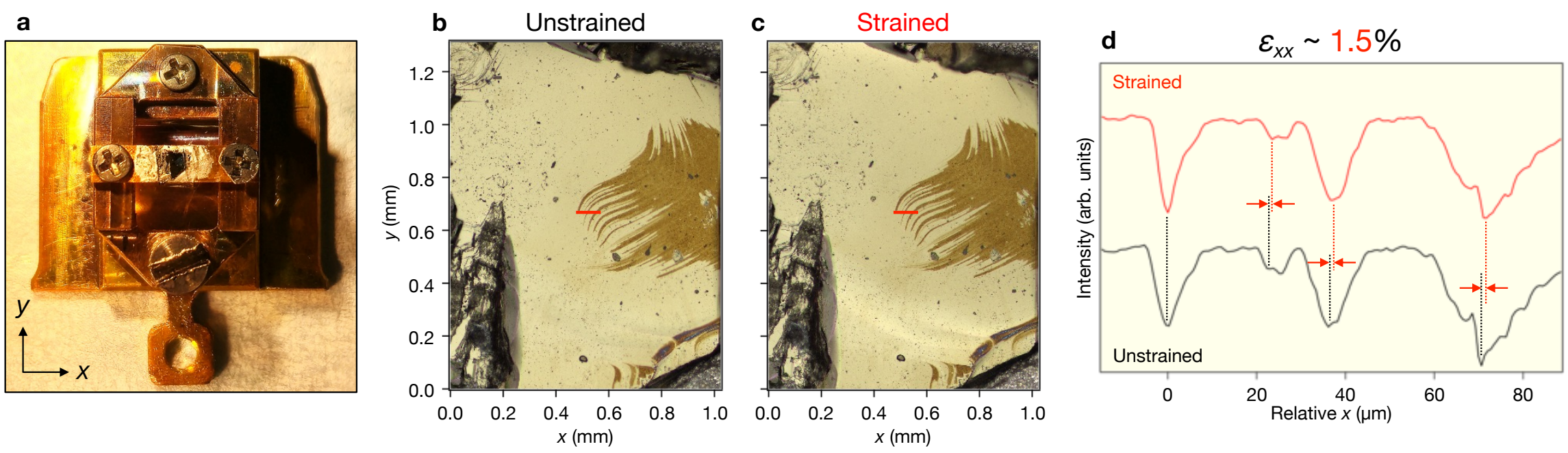

Supplementary Fig. 2 | Direct measurement of the surface in-plane strain by laser microscopy. **a** Photograph of the strain device. **b**, **c** Laser-microscope images of the sample surface for the unstrained and strained cases, respectively. **d** Intensity line profiles for the unstrained (black) and strained (red) cases along the red line in **b**,**c**, where the local in-plane strain ε_{xx} is evaluated.

stacking rearrangement.

Supplementary Note 2: Spatial map of electronic states

Supplementary Fig. 3 shows the near-E_F band structures measured at various different positions on the strain-applied sample with the width of ~0.5 mm. The results demonstrate uniform metallic behaviour throughout, confirming the robustness of the phase transition. These results support that the strain and the resulting electronic phase are essentially uniform within the probed area.

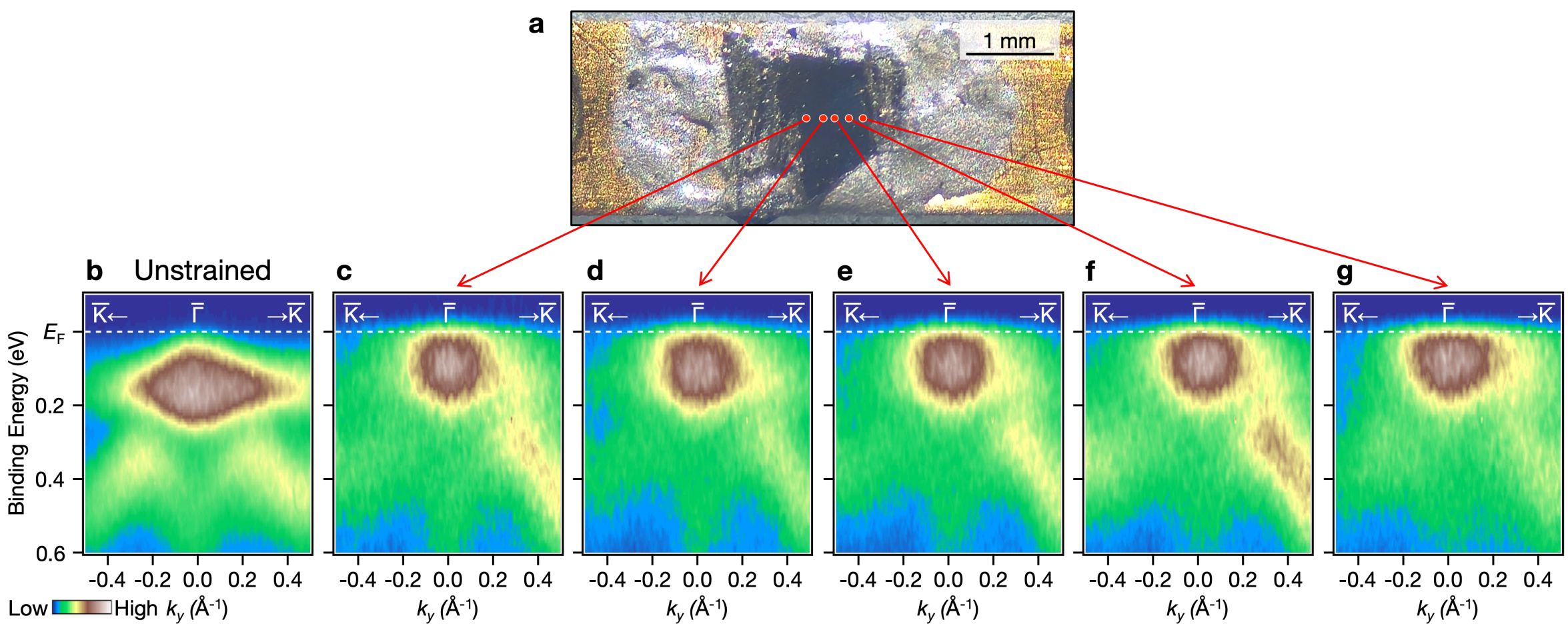

Supplementary Fig. 3 | Spatial homogeneity of the electronic states under applied strain. **a** Optical microscope image of the sample used for ARPES measurements. **b** ARPES-intensity plot measured along the $\overline{\Gamma K}$ cut measured at $h\nu$ = 103 eV for the unstrained case. **c-g** Same as **b**, but measured under tensile strain at various positions on the sample surface.

Supplementary Note 3: Reversibility of phase transition

After applying strain and performing the ARPES measurements, we released the strain and repeated the measurements. As shown in Supplementary Fig. 4, the characteristics of the electronic structure (such as the gap opening at E_F) after releasing strain are identical to those of the original unstrained phase, demonstrating that the strain-induced changes are fully

reversible. This confirms that the applied strain induces elastic deformation without causing irreversible structural damage or permanent changes to the electronic properties.

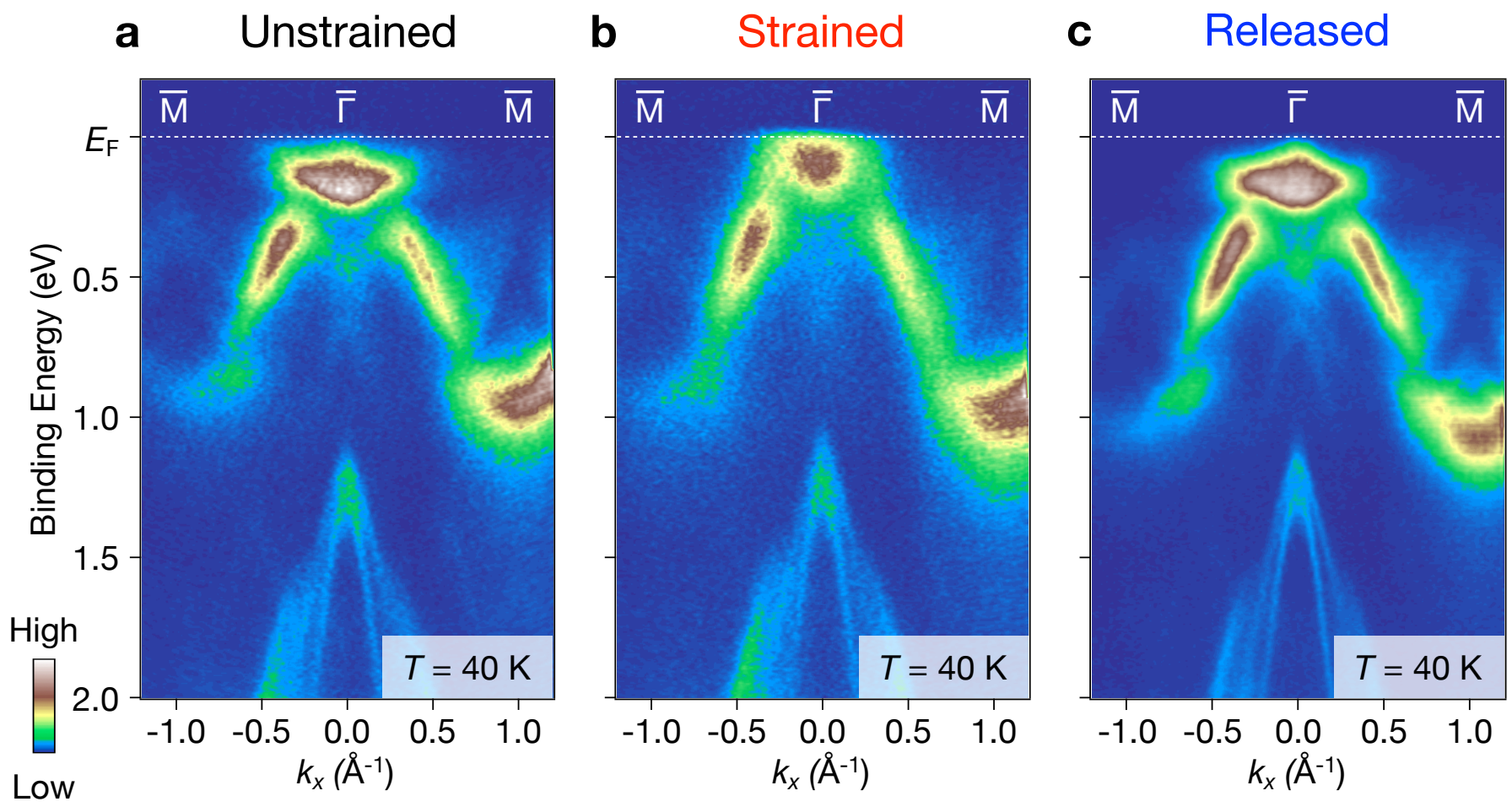

Supplementary Fig. 4 | Reversibility of the electronic structure. **a-c** ARPES-intensity plot measured along the $\overline{\Gamma \mathrm{M}}$ cut for the unstrained, tensile-strained, and strain-released conditions, respectively. The data demonstrate that the strain-induced metallic band structure in **b** completely reverts to the initial insulating state upon the release of the applied strain in **c**.

Supplementary Note 4: Evidence for the persistence of CDW under strain

Supplementary Fig. 5 shows the photoemission spectrum of the Ta 4f core levels in 1T-TaS_2 under uniaxial strain, measured at T = 40 K with photon energy $h\nu$ = 250 eV. One can identify the spin-orbit split components of the Ta 4$f_{5/2}$ and 4$f_{7/2}$ levels, each further splits into two peaks. According to a previous study[4], such splitting of the Ta 4f core levels is a hallmark of the formation of Star-of-David (SoD) clusters, reflecting the local lattice deformation and resulting inequivalent chemical bonding environments around Ta atoms. The present result thus provides strong evidence that the SoD clusters persist even under uniaxial strain.

Supplementary Fig. 6 displays ARPES intensity maps at a binding energy E_B = 1.25 eV as a function of two-dimensional wave vector, k_x, and k_y, for both unstrained and strained cases, measured at T = 40 K. In the unstrained sample (Supplementary Fig. 6a), a clear modulation of

ARPES intensity is observed, reflecting the periodicity of the reconstructed Brillouin zone (orange hexagons) due to the (√13×√13)R13.9° CDW. While the modulation is slightly weakened in the strained sample (Supplementary Fig. 6b), it remains discernible, indicating that the commensurate CDW persists even under strain.

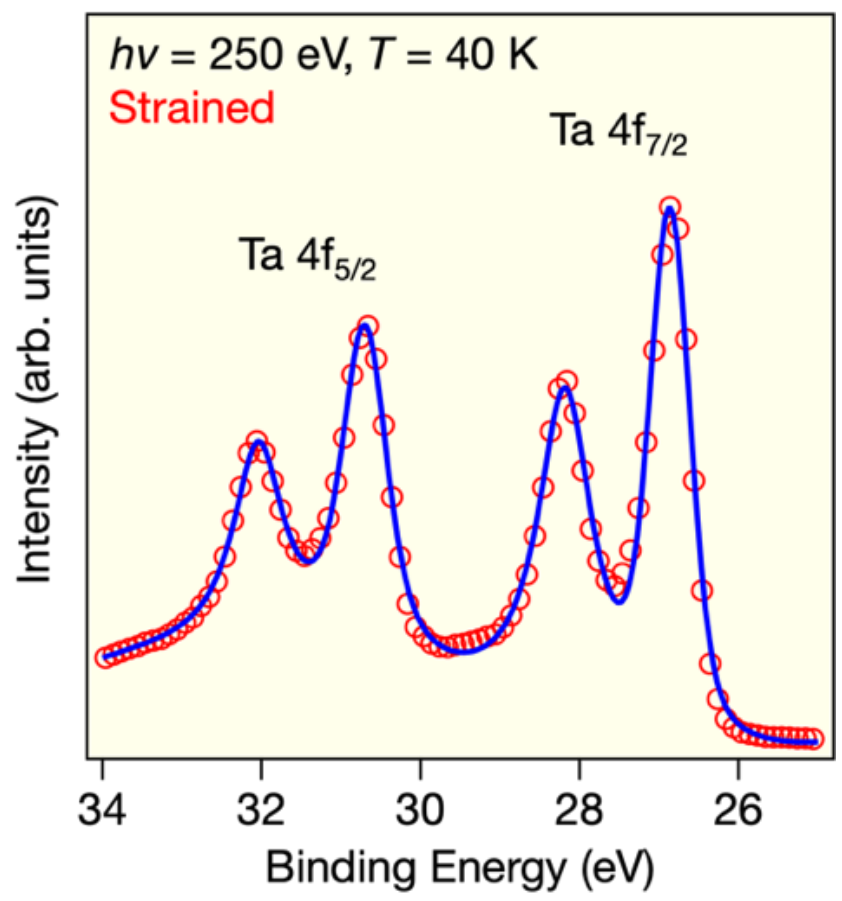

Supplementary Fig. 5 | Core-level splitting associated with the formation of SoD clusters. Ta 4*f* core-level photoemission spectrum measured under uniaxial strain at $T = 40$ K, together with a numerical fit (blue solid line) using four Lorentzian components and a moderate background. The observed splitting reflects inequivalent Ta sites arising from the formation of SoD clusters.

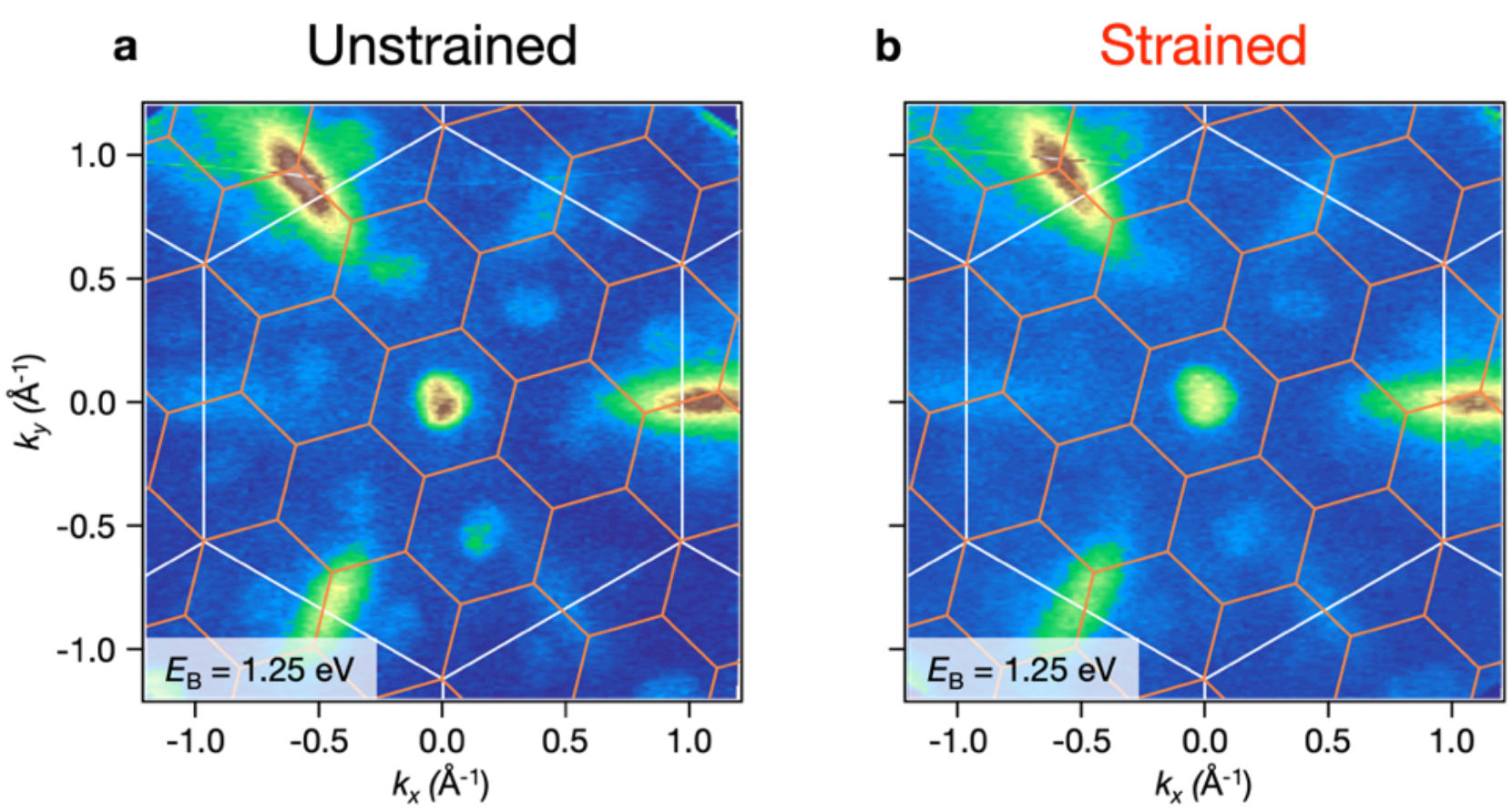

Supplementary Fig. 6 | Persistence of CDW-related periodic modulation in the strained phase. a, **b** ARPES-intensity maps at a binding energy $E_B = 1.25$ eV as a function of in-plane wave vectors k_x and k_y, measured at $T = 40$ K for the unstrained (a) and strained (b) phases, respectively. Orange hexagons indicate the folded Brillouin zone associated with the (√13×√13)R13.9° CDW.

Supplementary Note 5: Effect of shear strain

In our setup, bending the CuBe plate introduces strain to TaS_2. In this geometry, the applied strain is microscopically tensile but macroscopically shear. Because of the finite shear strain, if the TaS_2 layers were rigidly bonded, the maximum in-plane lattice expansion would occur at the topmost surface (Supplementary Fig. 7a). To minimize the large energy cost of such in-plane lattice distortion, the crystal would favorably relax the stress (Supplementary Fig. 7b). This can be viewed as a sort of interlayer slip and is expected to be most pronounced at the surface, so that it could lead to a surface-localized stacking transition that is also discussed in Supplementary Note 6.

Nevertheless, our case quantitatively differs from the strongly shear-strained case in heterostructure reported by Husremović et al.[5]. In their study, extremely strong shear is locally applied across a large step, leading to drastic atomic displacements involving the 1T-to-H structural transition. On the other hand, our macroscopic bending induces only a mild shear. Consequently, the in-plane 1T atomic structure is preserved in our experiments, and the weak shear acts merely as a perturbation on the electronic crystal (the SoD superlattice) to break the vertically aligned AL dimers.

It is noted that the present measurements alone do not allow us to exclude an intermediate partially slipped configuration. Such a partially slipped configuration may also involve local stacking faults that weaken the AL-type interlayer dimerization. Resolving the detailed atomic stacking would require direct structural characterization, for example by cross-sectional transmission electron microscopy, which is beyond the scope of the present work. As described in Supplementary Note 1, the representative ε_{zz} value is $\approx -0.4\%$, a scale at which the energy difference between the AL and L stackings is expected to be very small. Under such conditions,

even a relatively small shear perturbation induced by bending could facilitate a rearrangement toward an L-like stacking configuration.

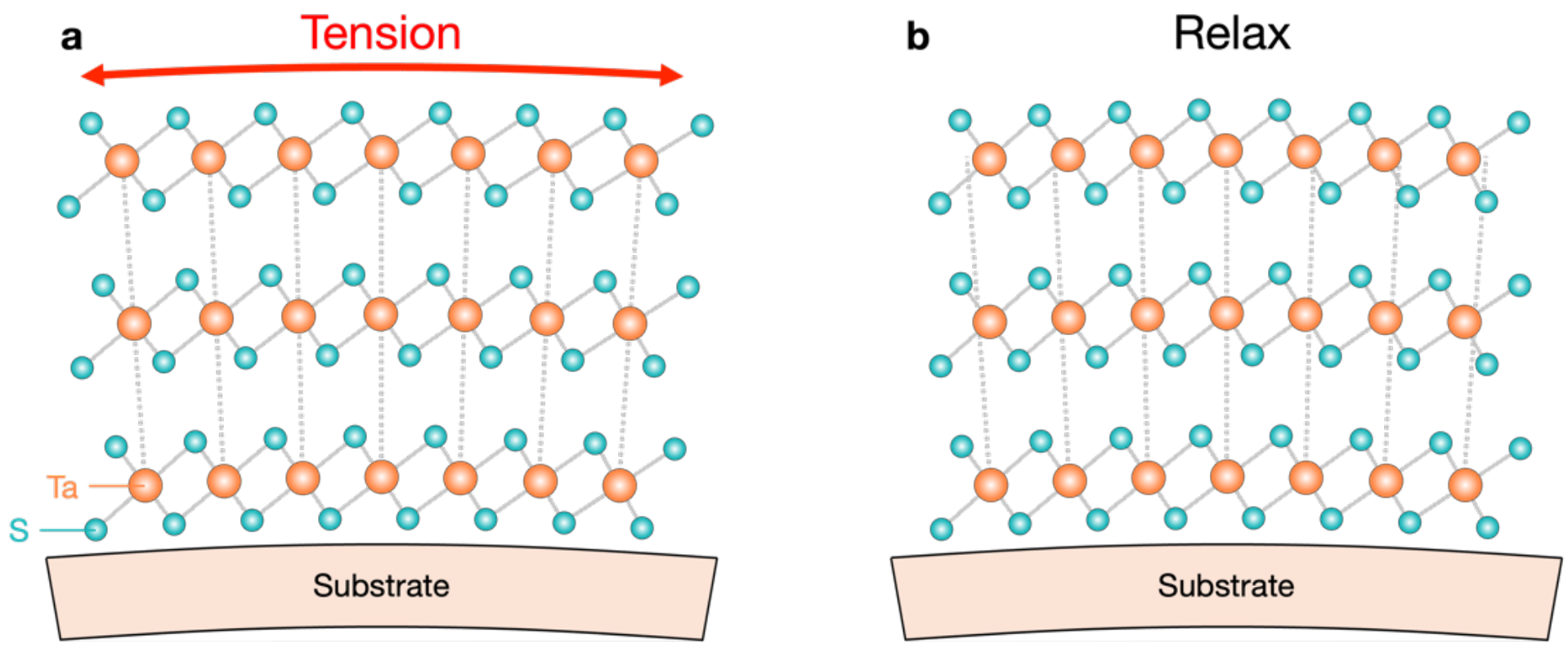

Supplementary Fig. 7 | Schematics of bent TaS_2 layers. a, **b** Schematics illustrating the bending induced by uniaxial strain in TaS_2 layers without and with assuming atomic relaxation.

Supplementary Note 6: XRD measurements of strained TaS_2

We have performed x-ray diffraction (XRD) measurements under uniaxial strain using the same device as in the ARPES measurements. The device was attached to a closed-cycle refrigerator. The experiments were conducted using a Rigaku 1.2-kW rotating anode x-ray generator with a VariMax-HF multilayer monochromator. The diffracted x-rays were collected by a two-dimensional XPAD S70 photon-counting pixel detector, which has an active area of 15 mm × 75 mm and a pixel size of 130 μm × 130 μm. The XRD results in Supplementary Fig. 8 obtained at $T = 50$ K (well below the CDW transition temperature of pristine TaS_2) show that the satellite peaks derived from the AL-stacked SoD structures (indicated by red arrows) remain visible even under strain, indicating that the AL stacking persists in the bulk crystal. This result suggests that the strain-induced stacking transition observed in ARPES primarily occurs locally at the surface, because ARPES dominantly probes the topmost layers of the crystal, whereas XRD reflects the bulk structure. This surface-selective transition is consistent

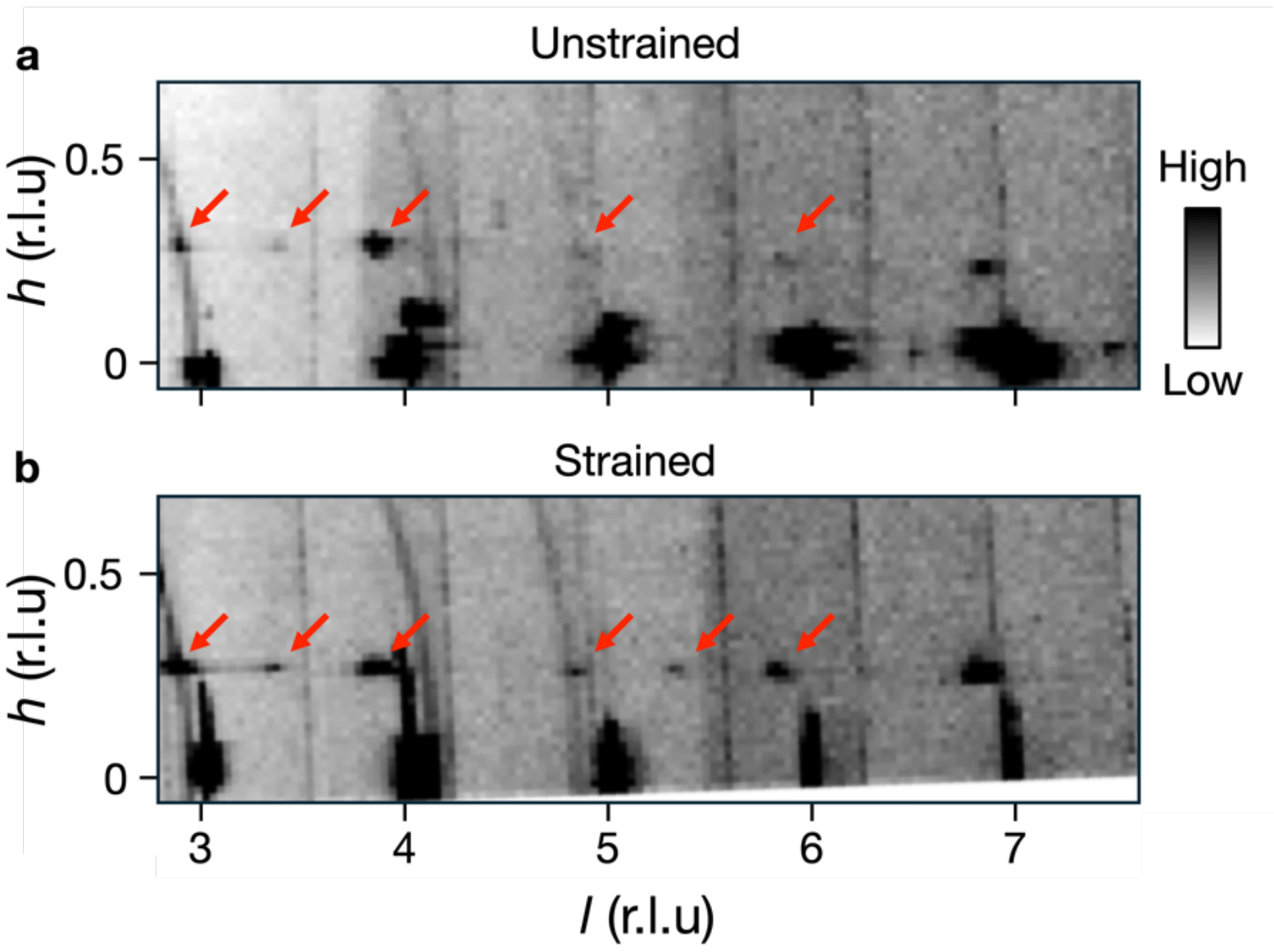

Supplementary Fig. 8 | Bulk crystal structure probed via x-ray diffraction. **a**, **b** Reciprocal space maps parallel to the *h0l*-plane for the unstrained and strained phases, respectively, at $T =$ 50 K.

with the shear-induced slip mechanism, which is most pronounced near the surface (Supplementary Note 5).

The photoelectron mean free path λ in our ARPES measurements at $h\nu$ = 98 eV is approximately 5 Å (ref. 6), comparable to the interlayer spacing of TaS_2 ($c = 5.92$ Å). Assuming that the n-th Ta layer is located at $z_n = (n - 1/2)c$ below the surface and that the photoemission intensity is attenuated as $\exp(-z_n/\lambda)$, we obtain attenuation factors of 0.553, 0.169, 0.052, and 0.016 for the first four Ta layers, corresponding to normalized contributions of 69.4%, 21.2%, 6.5%, and 2.0%, respectively, to the total ARPES signal. Thus, the topmost two Ta layers account for approximately 91% of the total ARPES signal. The absence of a clearly discernible insulating component in the strained ARPES spectra therefore suggests that the L-like reconstruction extends over at least the topmost two TaS_2 layers. Because the XRD probing depth is orders of magnitude larger than the thickness of a few TaS_2 layers, a thin reconstructed surface region would make a negligible contribution to the total diffraction intensity. The XRD

and ARPES results are therefore consistent with an L-like reconstruction in the near-surface layers that dominate the ARPES signal.

Supplementary Note 7: Comparison of band structure between strained metallic phase and NCCDW phase

One may question whether the strained metallic phase corresponds to the nearly commensurate CDW (NCCDW) phase, which typically appears above 180 K in the unstrained material. To

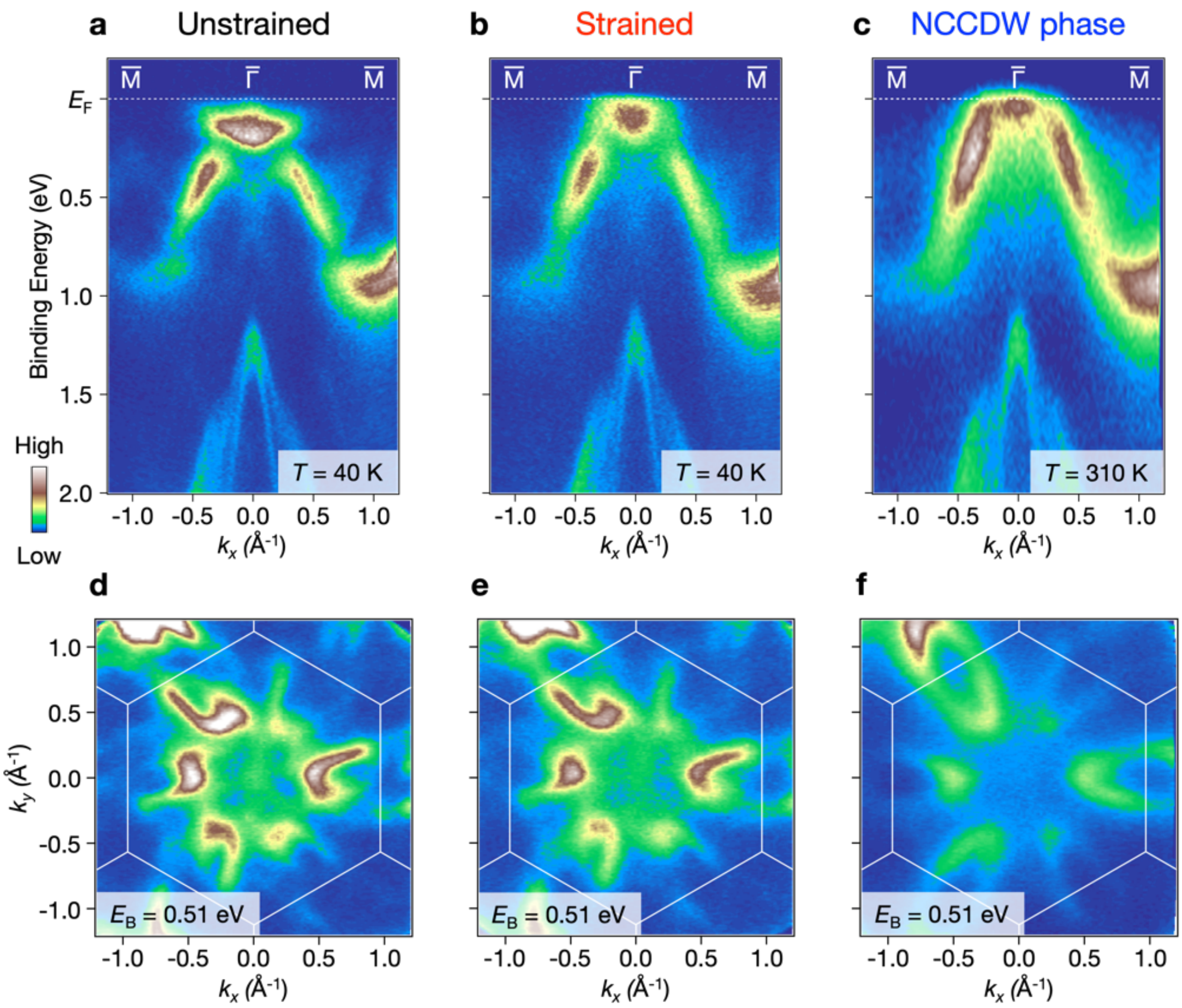

Supplementary Fig. 9 | Distinct spectral signatures of the strained phase and the NCCDW phase. a–c ARPES intensity along the $\overline{\Gamma M}$ cut in the unstrained phase at T = 40 K (a), the strained phase at T = 40 K (b), and the nearly commensurate CDW (NCCDW) phase at T = 310 K (c). **d–f** Corresponding ARPES-intensity maps at a binding energy of E_B = 0.51 eV. The strained and unstrained phases exhibit similar spectral features, including discontinuities and chiral patterns, which are absent in the NCCDW phase.

address this question, we compare in Supplementary Fig. 9a–c the ARPES intensity along the $\overline{\Gamma \mathrm{M}}$ cut among the unstrained phase, strained phase, and unstrained NCCDW phase. The spectral features of the unstrained and strained phases are largely similar, with a main difference being a weakening of the CDW gap and an increase in the near-E_F spectral weight in the strained case. In contrast, the NCCDW phase exhibits qualitatively different features: notably, the discontinuity in the spectral intensity at $E_B \sim 0.2$ eV and 0.8 eV, characteristic of the CDW-induced band hybridization, is almost absent, and a highly dispersive hole-like band clearly crosses E_F outside the electron pocket at $\overline{\Gamma}$. In addition, the chiral pattern in the ARPES-intensity maps observed in the unstrained and strained phases (Supplementary Fig. 9d,e) is nearly absent in the NCCDW phase (Supplementary Fig. 9f). These findings demonstrate that the strained phase is distinct from the NCCDW phase, despite both being metallic.

Supplementary Note 8: Comparison of two processes for ARPES measurements under uniaxial strain

We conducted ARPES measurements using two different procedures for applying uniaxial strain (Supplementary Fig. 10a). In Path 1, samples were strained ex situ, then loaded into ultrahigh vacuum (UHV), cooled to 40 K, cleaved in situ, and measured. In Path 2, samples were first loaded into UHV, cooled to 40 K, cleaved in situ, and initially measured without strain. Strain was then applied in situ, followed by further ARPES measurements. Supplementary Fig. 10c,d compares the ARPES intensity of the unstrained and strained phases obtained via both paths. The overall spectral features are highly consistent between the two methods, particularly with the presence of metallic spectral weight at E_F. This reproducibility confirms that the observed spectral features are intrinsic and not an artifact of the strain-application procedure.

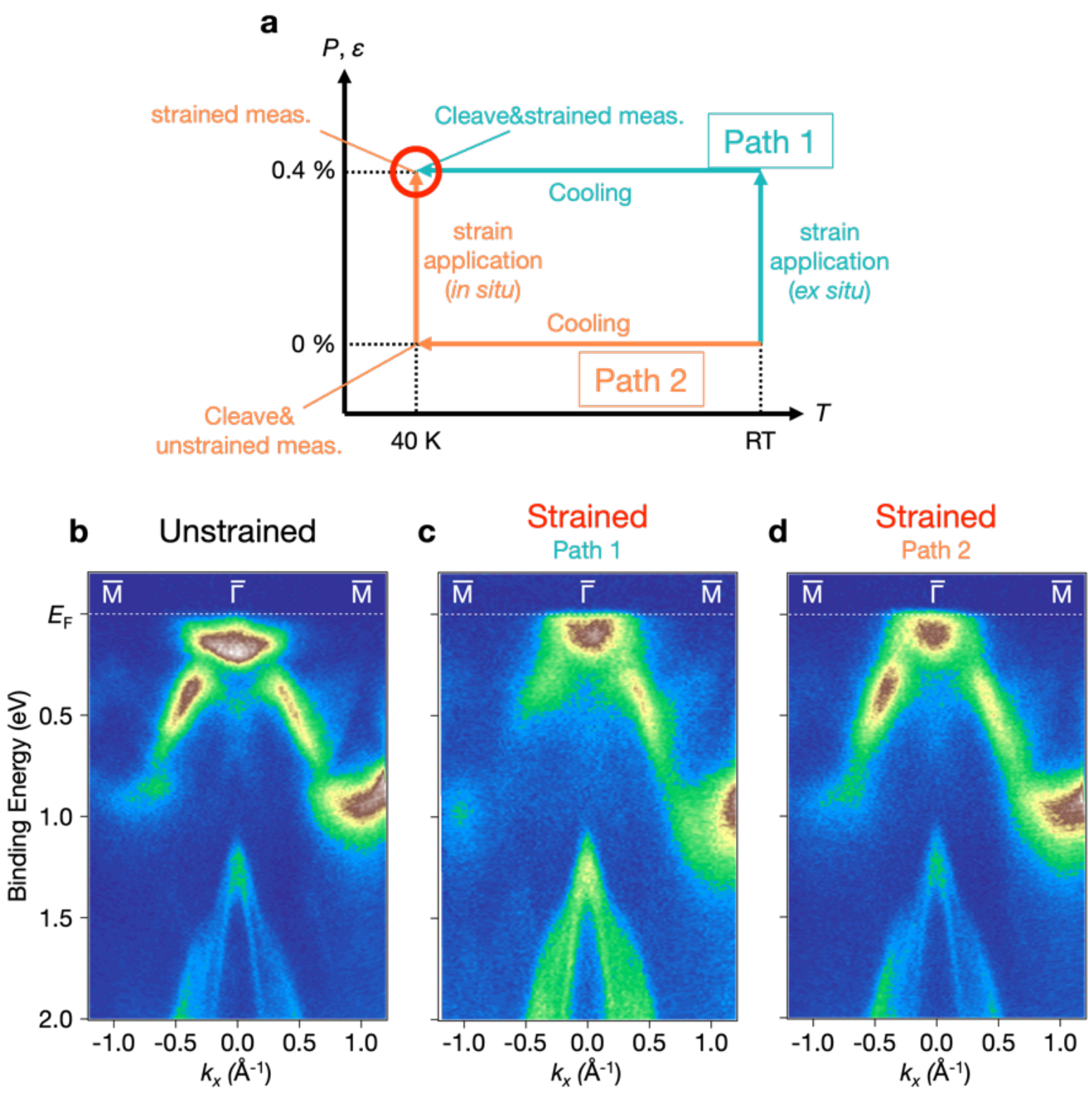

Supplementary Fig. 10 | Reproducibility of spectral features under different strain-application procedures. a Schematic illustration of two experimental paths used for ARPES measurements under uniaxial strain (Path 1: strain applied ex situ; Path 2: strain applied in situ). **b–d** ARPES intensity along the $\overline{\Gamma \mathrm{M}}$ cut measured at $T = 40$ K in the unstrained phase (b), the strained phase obtained via Path 1 (c), and the strained phase obtained via Path 2 (d). Consistent spectral features across both paths demonstrate the intrinsic nature of the strain-induced changes.

Supplementary References

1. Chernolevska, Y. et al. Strain-controlled superconductivity in epitaxially grown thin films of 1T-TaS_2. *Sci. Rep.* **15**, 36052 (2025).
2. Lee, S.-H., Goh, J. S. & Cho, D. Origin of the insulating phase and first-order metal-insulator transition in 1*T*-TaS_2. *Phys. Rev. Lett.* **122**, 106404 (2019).
3. Nicholson, C. W. et al. Gap collapse and flat band induced by uniaxial strain in 1*T*-TaS_2. *Phys. Rev. B* **109**, 035167 (2024).
4. Negishi, S. et al. Photoemission study on electronic structure of $TiSe_2$. *Physica B* **383**, 155 (2006).
5. Husremović, S. et al. Encoding multistate charge order and chirality in endotaxial heterostructures. *Nat. Commun.* **14**, 6031 (2023).
6. Seah, M. P. & Dench, W. A. Quantitative electron spectroscopy of surfaces: a standard data base for electron inelastic mean free paths in solids. *Surf. Interface Anal.* **1**, 2 (1979).
