# Supplementary material for: Strain-induced correlated metallic phase in 1T-TaS2
Source: Nat Commun. 2026 Nov 13;17:11407. doi: 10.1038/s41467-026-78883-1 (PMC13768348; doi:10.1038/s41467-026-78883-1)
Supplement: Supplementary file 2 [file 41467_2026_78883_MOESM2_ESM.pdf]

nature portfolio

Peer Review File

Strain-induced correlated metallic phase in 1T-TaS_2

Corresponding Author: Dr Kosuke Nakayama

This file contains all reviewer reports in order by version, followed by all author rebuttals in order by version.

Version 0:

Reviewer comments:

Reviewer #1

(Remarks to the Author)
This paper investigates how modest uniaxial strain converts the Mott-insulating 1T-TaS2 into a correlated metal. By integrating in-situ bridge actuation with high-resolution ARPES, the authors link the changes in interlayer stacking directly to the observed band structures. The main advance is the clear spectroscopic signature of a half-filled, renormalized band that crosses the Fermi level once the dimerization is fully suppressed in favor of single-layer L stacking. This study enriches our understanding of the underlying physics in the insulator-to-metal transition in TaS2 and correlated metallic phases.
The manuscript is clearly written, and the data is of high quality. Before publication, however, the following points should be addressed to solidify the stacking order and quantify the local strain.
1. The authors report tensile strain values “up to ~1%” extracted from finite element simulations and on-chip gauges, and probe the sample with micro-focused ARPES. Is the strain uniformly distribution in the sample?
2. In the low-temperature phase, each Star-of-David structure can adopt one of three symmetry-equivalent L sites giving rise to stacking disorder (Ritschel et al. PRB, 98, 195134 (2018) and Stahl et al. Nat. Commun. 11, 1247 (2020)). Does the applied tensile strain influence the random orientation or generate long-range order? Furthermore, related reports on bent graphene (Han et al. Nat. Mater. 19, 305 (2020)) and TaS2 heterostructure (Husremovic et al. Nat. Commun. 14, 6031 (2023)) show shear-induced slips. Have the authors searched for analogous shear in this strained structure?
3. The key evidence for dimer suppression is the agreement between ARPES spectra for the strained sample and the DFT band structure for ideal L stacking, with consistent results of nearly half-filled metallic band dispersion. Have the authors performed any bulk-sensitive measurements with high penetration depth such as synchrotron XRD, to verify that A-type bilayers are fully removed and that any residual A/L admixture lies below the detection limit? In a related study, Nicholson et al. (PRB, 109, 035167 (2024)) observe that uniaxial strain suppresses only part of the bilayer dimerization; XRD revealed a growing L-stacking shoulder while the main hybridization peak remained, and their ARPES spectra reveals a hybridization gap together with a -20 meV quasiparticle shoulder, consistent with an incomplete conversion to the L configuration.

Reviewer #2

(Remarks to the Author)
The study by S. Suzuki et al. investigates the strain-induced change of electronic band structure of 1T-TaS_2 using angle-resolved photoemission spectroscopy (ARPES) and first-principles calculations. The authors report an insulator-to-metal transition under uniaxial strain, evidenced by the closing of the electronic gap. By comparing ARPES spectra with theoretical calculations, they attribute the metallic phase to a strain-induced rearrangement of the charge-ordered interlayer stacking.

While I think the strain-induced modification of the band structure is overall interesting, the idea of this work is quite similar to Ref. 32 (PRB 109, 035167 2024), where uniaxial strain was similarly shown to reduce the electronic gap, and first-principles calculations combined with X-ray diffraction suggested a restructuring of the interlayer order. Notably, Ref. 32 systematically examined band structures under different stacking configurations and proposed the L stacking order as the structure in the strain-induced state.

In the present manuscript, the conclusion of the strain-induced metallic state with the L stacking in 1T-TaS2 is weakly estabilished by comparing the ARPES bands with the calculated results. The match between experiment and thoery is not very well, e.g. the large splitting of the bands in the energy range of 0.3-0.4 eV in the calculations is not observed in the ARPES bands.

It is not clear what the the strain distribution in the samples. Given the expected a continous strain gradient from the contact point, it remains questionable whether the probed region (even with micro-focused ARPES) exhibits the assumed uniform strain.

Therefore, there is no major advance in the understanding of either strain-induced band renormalization or the clear evidence on the interlayer stacking order in the strain-induced state. The novelty of this work is not sufficient to warrant publication in Nature Communications.

Reviewer #3

(Remarks to the Author)
In this work, Suzuki *et al*. report the combination of uniaxial tensile strain and ARPES measurements to explore the band structure of 1*T*-TaS_2. They observe a strain-induced insulating to metallic phase transition, which is attributed to a change in the stacking order, as supported by their DFT calculations. Furthermore, they identify strong electron correlation effects, evidenced by the change in the effective mass. Overall, the experimental observations are clear, the interpretations are generally self-consistent, and the manuscript is well-written. Given the relatively limited number of successful applications of tensile strain in ARPES studies, the results presented here are indeed novel and provide valuable information in understanding the complex phase transitions and the correlated nature in 1*T*-TaS_2. I recommend this work for publication in *Nature Communications*. Before the final recommendation, the following questions should be addressed to strengthen the manuscript.
1. The reversibility of strain-induced changes can sometimes be a concern in such studies. Can the system revert to its original unstrained phase upon release of the applied strain?
2. According to the DFT calculations, the CDW gap within E_B ranges of 0.7–0.9 eV increases when the system changes from AL to L stacking (Figs. 3e and 3f). However, experimentally, the spectral intensity within the CDW gap increases under strain, and the energy position of the gap edges remains fixed (Fig. 1k). Could the authors clarify this apparent discrepancy?
3. Since the application of tensile strain in this context is somehow technically innovative, more details should be provided regarding the characterization of strain. For example, the use of finite-element simulations and strain-gauges on page 6.
4. The DFT calculation details are essential, as they lay the foundation for interpreting changes in stacking order changes and band renormalization. These should be explicitly described in the Methods section and cannot be omitted.
5. On page 6, the term “confirming high single crystallinity of our sample” may be misleading. ARPES can reveal coherent band dispersion and a weak inelastic background, which support good crystallinity, but cannot definitively confirm single crystallinity from the observation of “several highly dispersive bands”.

Version 1:

Reviewer comments:

Reviewer #1

(Remarks to the Author)
The revised manuscript argues that the applied uniaxial tensile strain induces a surface-localized stacking rearrangement from an AL-like dimerized stacking toward an L-like, non-dimerized configuration. This interpretation is plausible, especially in view of the small energy difference between AL and L stackings discussed by Lee et al. PRL 122, 106404 (2019) and Nicholson et al. PRB 109, 035167 (2024). I would suggest the publication of the work. However, the manuscript would be strengthened to make a more quantitative connection between the externally applied bending strain and the proposed near-surface stacking and electronic reconstruction.

1. The authors discuss the possibility that the in-plane tensile strain reduces the out-of-plane interlayer distance through a Poisson response (lines 199-206 in the revised manuscript), and that the bending geometry may also induce a surface-enhanced shear and mild slip component. Could the authors estimate the actual strain tensor in the TaS2 crystal near the ARPES-probed surface, in particular the out-of-plane strain ε_zz and the relevant shear component, for the nominal applied ε_xx~1%?
This would be useful because previous works (Lee et al. & Nicholson et al.), suggested that AL and L stackings can become nearly degenerate for a reduction of the interlayer spacing of order ~0.3%. If the effective Poisson response of the present strained device leads to ε_zz in this range, the proposed AL-to-L tendency would be quantitatively supported. If not, it would be important to clarify whether the dominant driving mechanism is instead the surface-localized shear/slip relaxation.

2. The newly added XRD data show that AL-associated satellite peaks remain visible under strain, indicating that the bulk remains at least partially AL-like. Therefore, if the ARPES metallicity originates from a surface-localized L-like configuration, it would be helpful to estimate how many topmost layers are expected to undergo such a reconstruction.
Can the authors estimate how many topmost interlayer registries are expected to become L-like or partially slipped, and whether such a surface-confined region is sufficient to dominate the ARPES signal given the photoelectron escape depth?

3. The current discussion appears to compare mainly the two limiting cases of ideal AL stacking and ideal L stacking. However, the proposed bending-induced shear/slip mechanism may also produce an intermediate structure, in which the A-

stacked dimer is laterally displaced only partially. Such a partial slip could reduce the interlayer dimer hopping and decrease the dimerization gap, without necessarily reaching the ideal L-stacking registry.
Could the authors clarify whether the expected shear/slip is large enough to reduce A-stacking dimer hopping to an L-like value and fully close the dimerization gap? This would distinguish whether the observed metallic ARPES spectrum requires a true L-like surface registry, or whether it could arise from a partially slipped AL dimer with substantially weakened interlayer hybridization.

Reviewer #2

(Remarks to the Author)
This is my second round of review for this manuscript. The authors argue that they achieved a larger nominal strain than Ref. 32 (PRB 109, 035167 2024), but this is a quantitative rather than a qualitative difference and the observed evolution of the band structure—specifically the reduction of the electronic gap—follows the same trend already reported. The fact that the gap becomes smaller and eventually closes under larger strain is expected. Moreover, the ARPES data in the present manuscript are of poorer quality than those in Ref. 32 (band dispersion near the Fermi level is clearly observed with 6.2 eV laser ARPES in Ref. 32), making it difficult to confidently identify a clear insulator-to-metal transition. Furthermore, no dramatic change in band dispersion is observed across the transition.

Additionally, Figure 3 of the present manuscript bears a strong resemblance to Figure 3 of Ref. 32, both in terms of the stacking-dependent band structure and the overall conclusions.

Therefore, the claimed advances are incremental at best, and the manuscript lacks the conceptual or technical novelty required for publication in Nature Communications.

Reviewer #3

(Remarks to the Author)
I thank the authors for their careful revisions and detailed responses to my previous comments. All of my concerns have been satisfactorily addressed. In particular, the additional experiments have strengthened the paper. I believe the manuscript is now suitable for publication in Nature Communications and have no further requests for revision.

Version 2:

Reviewer comments:

Reviewer #1

(Remarks to the Author)
All my questions are properly answered by the authors. I support the publication of this manuscript in the current form.

Reviewer #2

(Remarks to the Author)
I have read the authors' reply and the revised manuscript. However, my primary concern regarding the novelty of the work remains unaddressed. The present study relies on an identical experimental setup and the sample system as those detailed in Ref. 32 (Supplementary Methods). The only distinguishable difference is the authors' assertion that applying higher strain drives the system into a "metallic" state, which they appear to acknowledge. This metallicity is supported solely by a shift in the spectral edge, a phenomenon that was already observed, albeit to a lesser extent, in Ref. 32.

The XRD results directly contradict the ARPES-based conclusion. The authors attempt to resolve this by invoking an L-like reconstruction in the near-surface layers, while asserting that AL stacking persists in the bulk. This interpretation, however, rests on conjecture.In fact, a more straightforward explanation is that the ARPES signal originates from strain-induced lattice constant change, stacking faults and/or disorder, which would naturally produce the observed spectral shifts.

Open Access This Peer Review File is licensed under a Creative Commons Attribution 4.0 International License, which permits use, sharing, adaptation, distribution and reproduction in any medium or format, as long as you give appropriate credit to the original author(s) and the source, provide a link to the Creative Commons license, and indicate if changes were made.
In cases where reviewers are anonymous, credit should be given to 'Anonymous Referee' and the source.
The images or other third party material in this Peer Review File are included in the article's Creative Commons license, unless indicated otherwise in a credit line to the material. If material is not included in the article's Creative Commons license and your intended use is not permitted by statutory regulation or exceeds the permitted use, you will need to obtain permission directly from the copyright holder.
To view a copy of this license, visit https://creativecommons.org/licenses/by/4.0/

Response to referees:

We sincerely thank all the referees for their careful evaluation of our manuscript and for the constructive comments and suggestions. We have revised the manuscript accordingly and believe that the revisions have significantly improved the clarity and completeness of the work. Below we provide a detailed point-by-point response to the referees' comments.

To the comments from the First Referee:

Referee comment: *This paper investigates how modest uniaxial strain converts the Mott-insulating 1T-TaS_2 into a correlated metal. By integrating in-situ bridge actuation with high-resolution ARPES, the authors link the changes in interlayer stacking directly to the observed band structures. The main advance is the clear spectroscopic signature of a half-filled, renormalized band that crosses the Fermi level once the dimerization is fully suppressed in favor of single-layer L stacking. This study enriches our understanding of the underlying physics in the insulator-to-metal transition in TaS_2 and correlated metallic phases.*

The manuscript is clearly written, and the data is of high quality. Before publication, however, the following points should be addressed to solidify the stacking order and quantify the local strain.

Our response: We thank the referee for the positive assessment and the constructive comments. We have carefully considered your comments and thoroughly revised the manuscript.

Referee comment: *1. The authors report tensile strain values "up to ~1%" extracted from finite element simulations and on-chip gauges, and probe the sample with micro-focused ARPES. Is the strain uniformly distribution in the sample?*

Our response: We thank the referee for this question. The strain can be regarded as uniform within the region probed by ARPES, as described in the following.

According to our finite-element-method simulations assuming a sample with realistic dimensions of 1×1×0.05 mm^3 glued on a CuBe plate (Fig. R1, reproduced from Fig. 1e), the strain macroscopically varies from ~1% at the center of the sample to ~0.2% near the edges, corresponding to a gradient of 1.6%/mm. Within the ~12-μm width of the beam spot in our micro-ARPES system (see red dot), this gradient corresponds to a strain variation of only ~0.02%, which is two orders of magnitude smaller than the applied

maximum strain. Therefore, the strain variation is negligible in our micro-ARPES measurements.

We have described this point in the revised manuscript (p. 6, lines 116–119) and updated the inset of Fig. 1e.

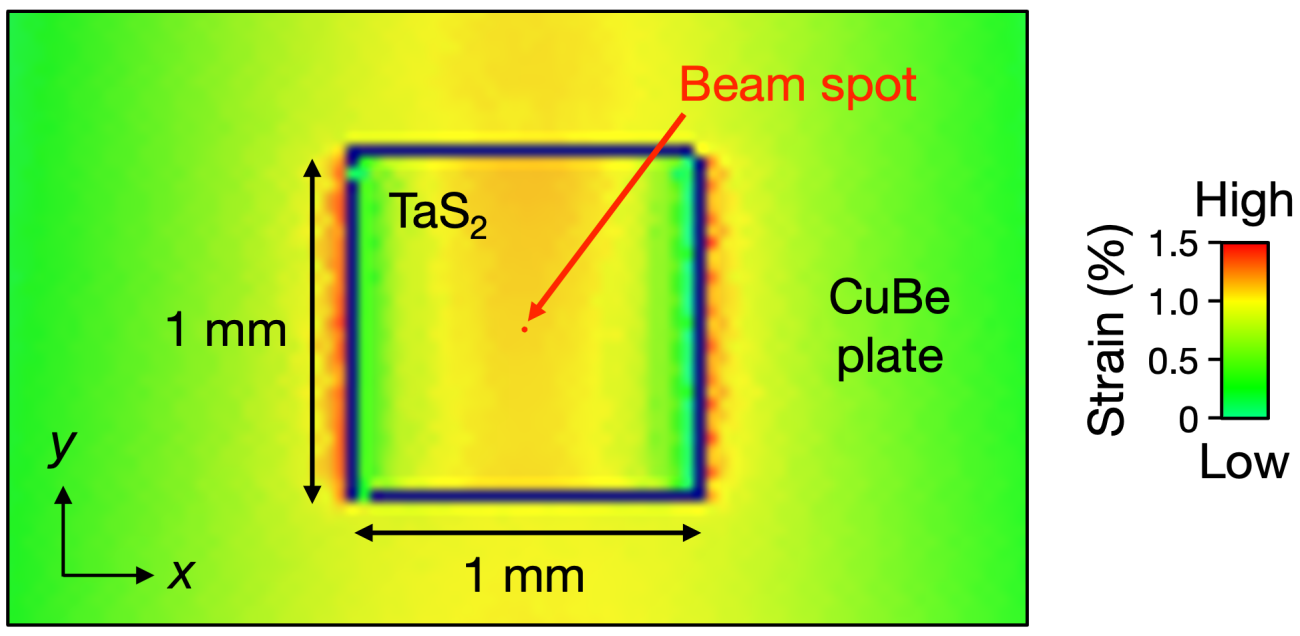

Fig. R1 | Spatial distribution of tensile strain simulated using the finite element method. Plot of ε_{xx} values calculated for a 1×1×0.05 mm^3 sample glued on CuBe plate.

Referee comment: *2. In the low-temperature phase, each Star-of-David structure can adopt one of three symmetry-equivalent L sites giving rise to stacking disorder (Ritschel et al. PRB, 98, 195134 (2018) and Stahl et al. Nat. Commun. 11, 1247 (2020)). Does the applied tensile strain influence the random orientation or generate long-range order?*

Our response: We thank the referee for this question. As reported by Ritschel *et al*. and Stahl *et al*. (refs. 16, 31), the existence of three symmetry-equivalent L sites leads to stacking disorder in the absence of strain. On the other hand, under uniaxial strain, it is plausible that the three-fold in-plane rotational symmetry of the lattice is broken, thereby lifting the degeneracy of the three L sites. The uniaxial strain would thus selectively suppress the random orientations and promote macroscopic long-range stacking order. We have described this point in the revised manuscript (p. 11, lines 214–219).

Referee comment: *Furthermore, related reports on bent graphene (Han et al. Nat. Mater. 19, 305 (2020)) and TaS_2 heterostructure (Husremovic et al. Nat. Commun. 14, 6031 (2023)) show shear-induced slips. Have the authors searched for analogous shear in this strained structure?*

Our response: We thank the referee for this thoughtful comment. As the referee pointed out, the interlayer shear, cooperatively with the Coulomb repulsion between the interlayer SoD lattices, could trigger the stacking transition in our strained TaS_2.

Specifically, in our setup, bending the CuBe plate introduces strain to TaS_2. In this geometry, the applied strain is microscopically tensile but macroscopically shear. Because of the finite shear strain, if the TaS_2 layers were rigidly bonded, the maximum in-plane lattice expansion would occur at the topmost surface (Fig. R2a). To minimize the large energy cost of such in-plane lattice distortion, the crystal would favorably relax the stress (Fig. R2b). This can be viewed as a sort of interlayer slip and is expected to be most pronounced at the surface, so that it could lead to a surface-localized stacking transition that will be discussed in detail in our response to the referee's comment 3.

Nevertheless, our case quantitatively differs from the model proposed by Husremovic *et al*. In their study, extremely strong shear is locally applied across a large step, leading to drastic atomic displacements involving the 1T-to-H structural transition. On the other hand, our macroscopic bending induces only a mild shear. Consequently, the in-plane 1T atomic structure is preserved in our experiments, and the weak shear acts merely as a perturbation on the electronic crystal (the SoD superlattice) to break the vertically aligned AL dimers.

We have described these points in the revised manuscript (p. 10, lines 207–210) and new Supplementary note 5, and cited the previous works by Husremovic *et al*. and Han *et al*. as new refs. 64 and 65.

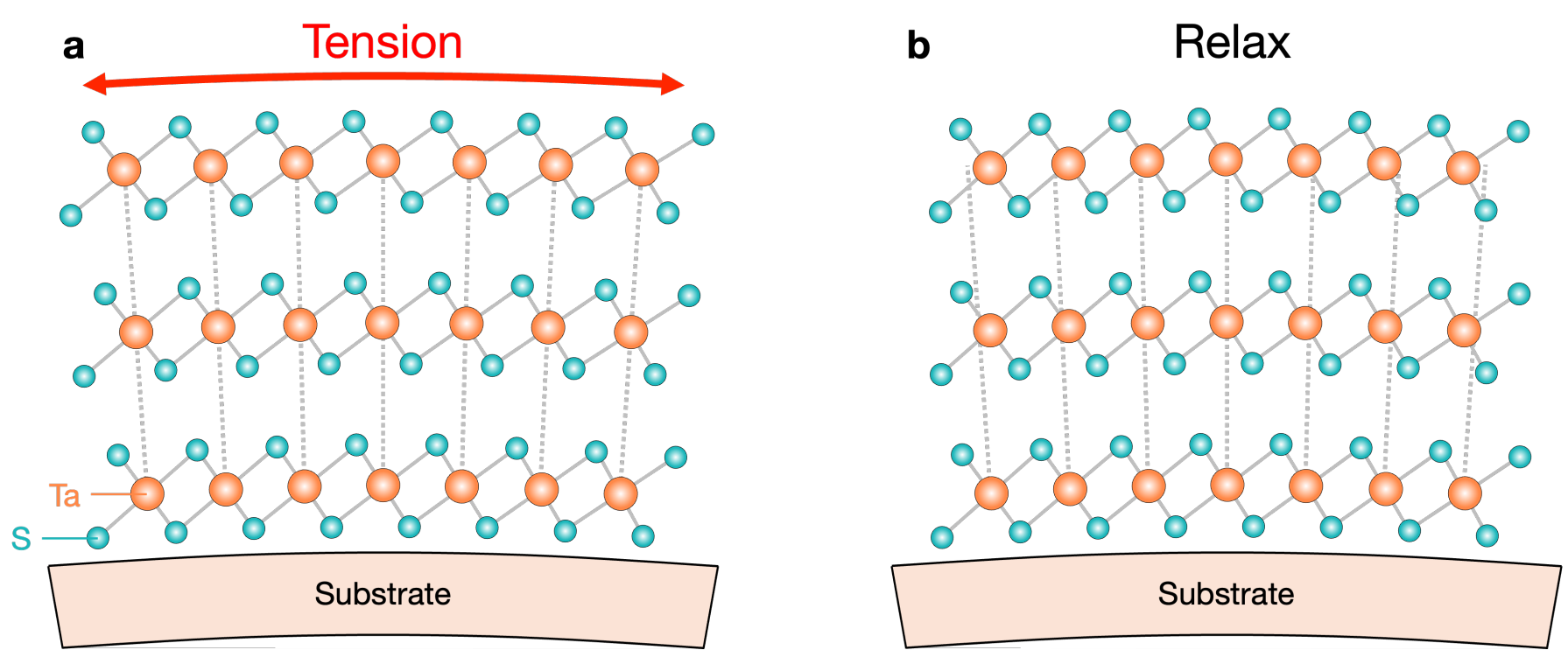

Fig. R2 | Schematics of bent TaS_2 layers. **a**, **b** Schematics illustrating the bending induced by uniaxial strain in TaS_2 layers without and with assuming atomic relaxation.

Referee comment: *3. The key evidence for dimer suppression is the agreement between ARPES spectra for the strained sample and the DFT band structure for ideal L stacking, with consistent results of nearly half-filled metallic band dispersion. Have the authors performed any bulk-sensitive measurements with high penetration depth such as synchrotron XRD, to verify that A-type bilayers are fully removed and that any residual A/L admixture lies below the detection limit? In a related study, Nicholson et al. (PRB, 109, 035167 (2024)) observe that uniaxial strain suppresses only part of the bilayer dimerization; XRD revealed a growing L-stacking shoulder while the main hybridization peak remained, and their ARPES spectra reveals a hybridization gap together with a -20 meV quasiparticle shoulder, consistent with an incomplete conversion to the L configuration.*

Our response: We thank the referee for raising an important point. To address this comment, we have newly performed XRD measurements under uniaxial strain using the same device as in the ARPES measurements. The XRD results in Fig. R3 obtained at T = 50 K (well below the CDW transition temperature of pristine TaS_2) show that the satellite peaks originating from the AL-stacked SoD structures (indicated by red arrows) remain visible even under strain, indicating that the AL stacking persists in the bulk crystal.

This result suggests that the strain-induced stacking transition observed in ARPES primarily occurs locally at the surface because ARPES dominantly probes the topmost

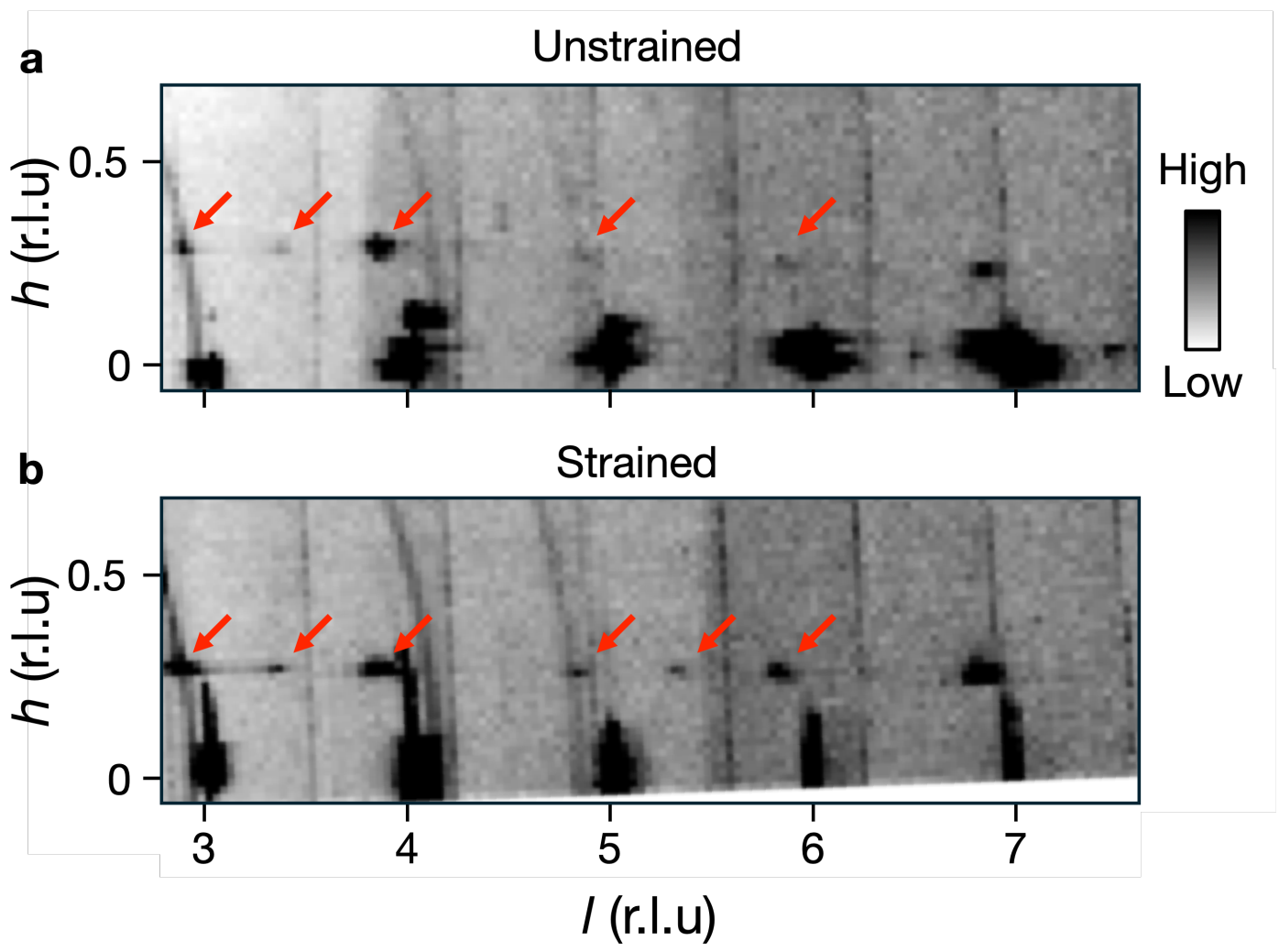

Fig. R3 | Bulk crystal structure probed via x-ray diffraction. **a**, **b** Reciprocal space maps parallel to the *h0l*-plane for the unstrained and strained phases, respectively, obtained at T = 50 K.

layers of the crystal, whereas XRD reflects the spatially averaged bulk structure. This surface-selective transition is consistent with the aforementioned shear-induced slip mechanism, which is most pronounced near the surface. Therefore, the present ARPES results support the strain-driven stacking transition at the surface limit, even if the structural transition is not uniformly realized throughout the bulk crystal.

We have described the surface-selective character in the revised manuscript (pp. 10–11, lines 210–211) and included the XRD data as new Supplementary note 6.

To the comments from the Second referee:

Referee comment: *The study by S. Suzuki et al. investigates the strain-induced change of electronic band structure of 1T-TaS_2 using angle-resolved photoemission spectroscopy (ARPES) and first-principles calculations. The authors report an insulator-to-metal transition under uniaxial strain, evidenced by the closing of the electronic gap. By comparing ARPES spectra with theoretical calculations, they attribute the metallic phase to a strain-induced rearrangement of the charge-ordered interlayer stacking.*

Our response: We thank the referee for the careful evaluation of our work and for the constructive comments. Below we address each point in detail.

Referee comment: *While I think the strain-induced modification of the band structure is overall interesting, the idea of this work is quite similar to Ref. 32 (PRB 109, 035167 2024), where uniaxial strain was similarly shown to reduce the electronic gap, and first-principles calculations combined with X-ray diffraction suggested a restructuring of the interlayer order. Notably, Ref. 32 systematically examined band structures under different stacking configurations and proposed the L stacking order as the structure in the strain-induced state.*

Our response: We thank the referee for highlighting the comparison with Ref. 32. However, we respectfully disagree with the referee's assessment that the novelty of our work is limited. Our study advances beyond Ref. 32 in two key aspects: (i) the realization of a strain-induced metallic phase and (ii) the direct observation of strong correlation effects in this metallic state.

Regarding (i), as we already explicitly noted in our original manuscript, Ref. 32 reported that uniaxial strain of ~0.2% reduces the insulating gap, but the system remains

gapped. Indeed, in Ref. 32, the band approaches the Fermi level but does not cross it. In stark contrast, by applying a larger strain of ~1%, we drive the system across a genuine insulator-to-metal transition. This is unambiguously evidenced by the closing of the gap and the emergence of a Fermi surface. Since insulating and metallic phases are fundamentally distinct electronic phases, the realization of a strain-induced metallic state constitutes a qualitatively new regime compared with the previous study.

Regarding (ii), the stabilization of the metallic phase enables us to analyze the quasiparticle dispersion and evaluate correlation effects for the first time. We observe a pronounced bandwidth renormalization corresponding to an effective mass enhancement factor of 2, demonstrating that the strain-induced phase is a strongly correlated metal. Such information could not be obtained in Ref. 32 because the system remained gapped.

Therefore, while both studies investigate strain effects in 1T-TaS_2, our first experimental realization of a strain-induced correlated metallic phase is a major advance, offering new insight into the interplay between interlayer stacking and electron correlation in this material. We have clarified these distinctions in the revised manuscript (pp. 11–12, lines 227–232).

Referee comment: *In the present manuscript, the conclusion of the strain-induced metallic state with the L stacking in 1T-TaS_2 is weakly estabilished by comparing the ARPES bands with the calculated results. The match between experiment and thoery is not very well, e.g. the large splitting of the bands in the energy range of 0.3-0.4 eV in the calculations is not observed in the ARPES bands.*

Our response: We thank the referee for this comment. Overall, we find good agreement between the ARPES spectra and the theoretical calculations, particularly regarding the Fermi surface topology and the half-filled band character, both of which support the L-stacking configuration.

While the referee correctly pointed out the invisibility of the large band splitting that is predicted in the energy range of 0.3-0.4 eV, this absence does not necessarily contradict the L-stacking scenario. Rather, it most likely reflects the strongly suppressed spectral intensity of the predicted bands; because they correspond to folded bands originating from the large superlattice, their photoemission cross-section is intrinsically weak. This intensity suppression, combined with inevitable spectral broadening in ARPES measurements, makes the splitting difficult to resolve. This is evident from the weak

intensity of the folded bands indicated by the yellow arrows in Fig. R4a, as well as the overall broadening of spectral features in the experimental ARPES data (Fig. R4c). This interpretation is further supported by an analogous situation in the unstrained pristine phase. Specifically, although band calculations for the AL stacking predict a similar splitting of folded bands in the same energy range (Fig. R4b), this splitting is not clearly resolved in the ARPES spectra (Fig. R4c). Therefore, the absence of the predicted band splitting is most likely due to the weak photoemission intensity of these folded bands and experimental spectral broadening, rather than a fundamental discrepancy between experiment and theory.

We have described this point in the revised manuscript (p. 10, lines 196–199).

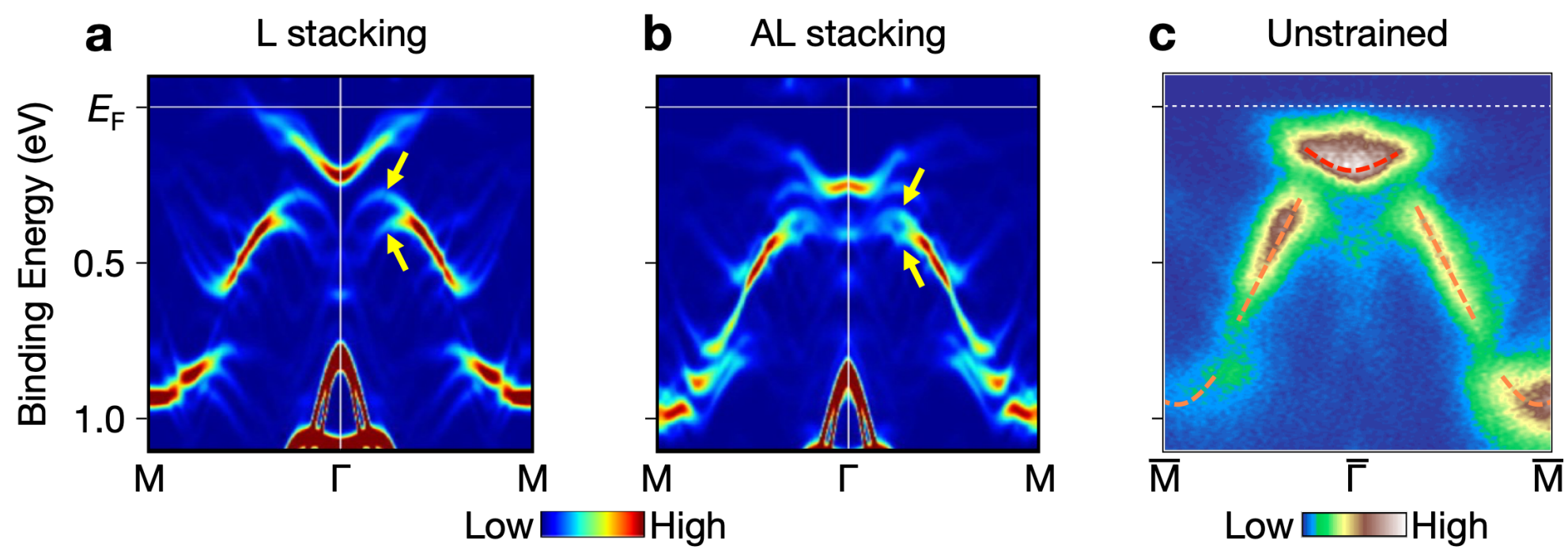

Fig. R4 | Comparison of calculated and experimental band structures. **a**, **b** Calculated electronic structures for L and AL stacking configurations respectively, reproduced from Fig. 3e,f of the main text. **c** ARPES intensity of 1T-TaS_2 for the unstrained phase, reproduced from Fig. 1h of the main text.

<u>Referee comment</u>: *It is not clear what the the strain distribution in the samples. Given the expected a continous strain gradient from the contact point, it remains questionable whether the probed region (even with micro-focused ARPES) exhibits the assumed uniform strain.*

<u>Our response</u>: We thank the referee for this question. The strain can be regarded as uniform within the region probed by ARPES, as described in the following.

According to our finite-element-method simulations assuming a sample with realistic dimensions of 1×1×0.05 mm^3 glued on a CuBe plate (Fig. R5, reproduced from Fig. 1e), the strain macroscopically varies from ~1% at the center of the sample to ~0.2% near the edges, corresponding to a gradient of 1.6%/mm. Within the ~12-μm width of the beam spot in our micro-ARPES system (see red dot), this gradient corresponds to a strain

variation of only ~0.02%, which is two orders of magnitude smaller than the applied maximum strain. Therefore, the strain variation is negligible in our micro-ARPES measurements. We have described this point in the revised manuscript (p. 6, lines 116–119).

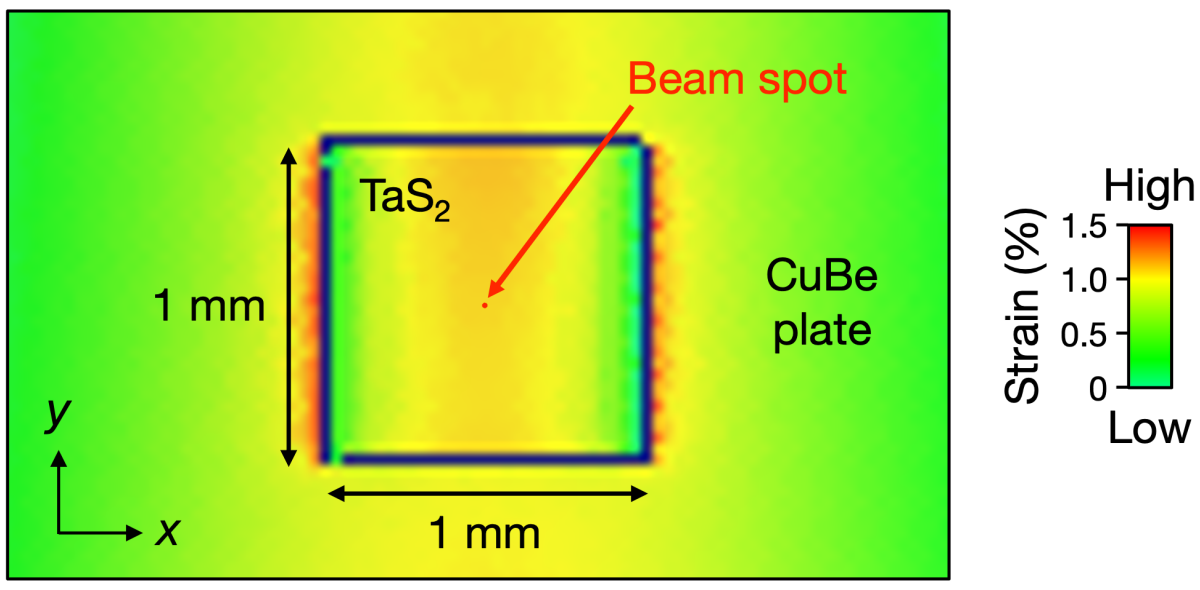

Fig. R5 | Spatial distribution of tensile strain simulated using the finite element method. Plot of ε_{xx} values calculated for a 1×1×0.05 mm^3 sample glued on CuBe plate.

We have also confirmed spatial homogeneity of the electronic states. Figure R6 shows the near-E_F band structures measured at various different positions on the strain-applied sample with the width of ~0.5 mm. The results demonstrate uniform metallic behavior throughout, confirming the robustness of the phase transition. These results support that the strain and the resulting electronic phase are essentially uniform within the probed area.

We have described these points in the revised manuscript (p. 7, line 145) and in new Supplementary note 2.

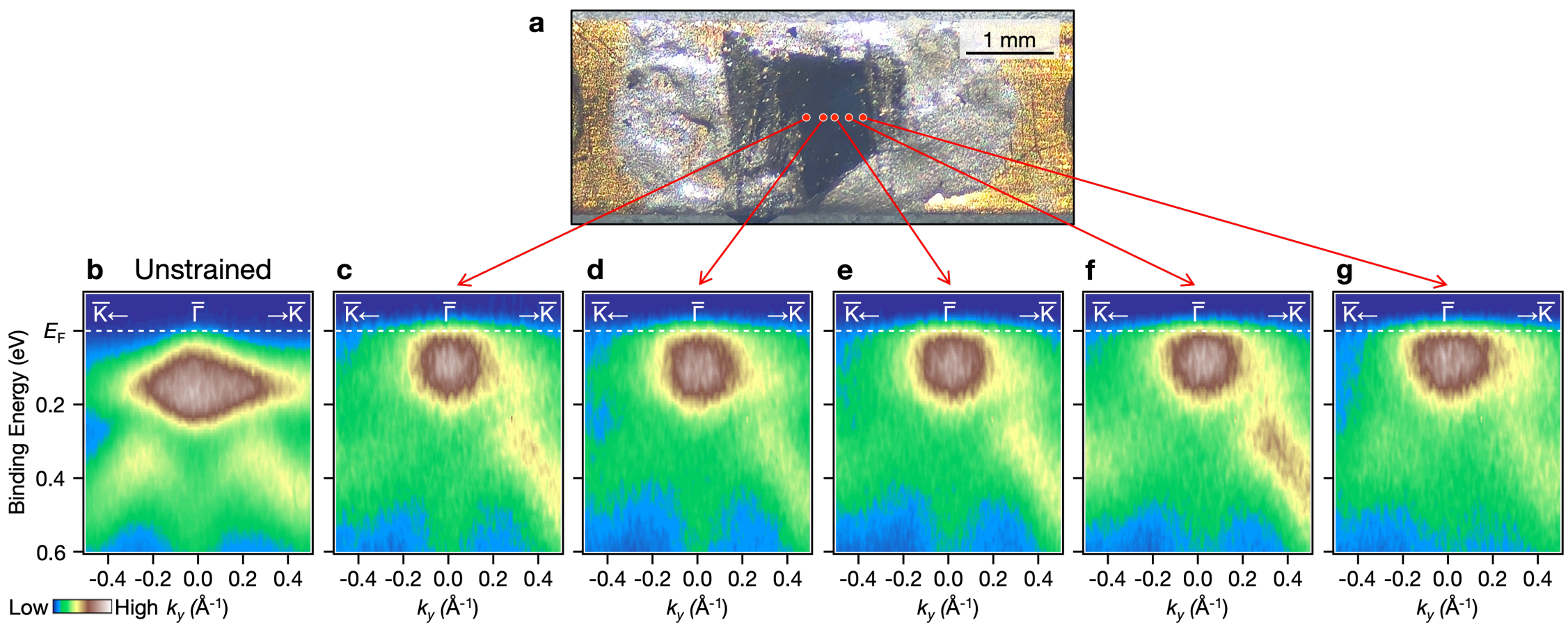

Fig. R6 | Spatial homogeneity of the electronic states under applied stain. **a** Optical microscope image of the sample used for ARPES measurements. **b** ARPES-intensity plot measured along the $\overline{\Gamma K}$ cut measured at $h\nu$ = 103 eV for the unstrained case. **c-g** Same as **b**, but measured under tensile strain at various positions on the sample surface.

Referee comment: *Therefore, there is no major advance in the understanding of either strain-induced band renormalization or the clear evidence on the interlayer stacking order in the strain-induced state. The novelty of this work is not sufficient to warrant publication in Nature Communications.*

Our response: As described above, our work for the first time establishes a genuine strain-induced insulator-to-metal transition and reveals the strongly correlated nature of the resulting metallic state.

Even if some quantitative differences remain between the experimental spectra and the calculations, the key experimental observations (i.e., the gap closure, the emergence of a half-filled Fermi surface, and the strong bandwidth renormalization) are robust.

For these reasons, we believe that the present work represents a significant advance beyond previous studies and provides new insight into strain-controlled electronic phases in layered quantum materials.

To the comments from the Third referee:

Referee comment: *In this work, Suzuki et al. report the combination of uniaxial tensile strain and ARPES measurements to explore the band structure of 1T-TaS_2. They observe a strain-induced insulating to metallic phase transition, which is attributed to a change in the stacking order, as supported by their DFT calculations. Furthermore, they identify strong electron correlation effects, evidenced by the change in the effective mass. Overall, the experimental observations are clear, the interpretations are generally self-consistent, and the manuscript is well-written. Given the relatively limited number of successful applications of tensile strain in ARPES studies, the results presented here are indeed novel and provide valuable information in understanding the complex phase transitions and the correlated nature in 1T-TaS_2. I recommend this work for publication in Nature Communications. Before the final recommendation, the following questions should be addressed to strengthen the manuscript.*

Our response: We thank the referee for the positive evaluation of our work and for the helpful suggestions to further improve the manuscript. We have revised the manuscript following the referee' suggestions.

Referee comment: *1. The reversibility of strain-induced changes can sometimes be a*

concern in such studies. Can the system revert to its original unstrained phase upon release of the applied strain?

Our response: We thank the referee for this important question. We have confirmed that the phase transition is reversible. After applying strain and performing the ARPES measurements, we released the strain and repeated the measurements. As shown in Fig. R7, the characteristics of the electronic structure (such as the gap opening at E_F) after releasing strain are identical to those of the original unstrained phase, demonstrating that the strain-induced changes are fully reversible. This confirms that the applied strain induces elastic deformation without causing irreversible structural damage or permanent changes to the electronic properties. We have included this result in new Supplemental note 3.

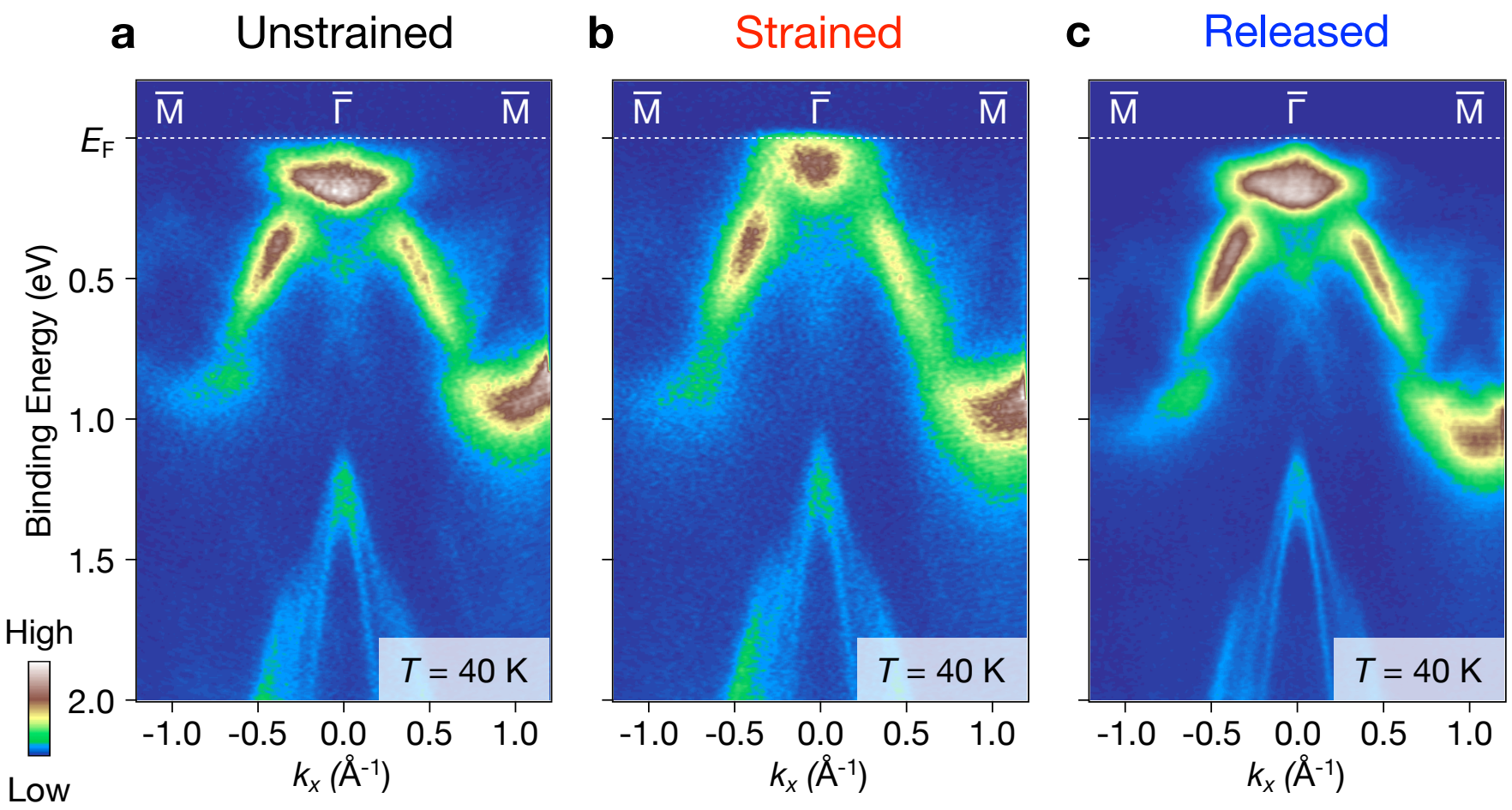

Fig. R7 | Reversibility of the electronic structure. **a-c** ARPES-intensity plot measured along the $\overline{\Gamma M}$ cut for the unstrained, tensile-strained, and strain-released conditions, respectively. The data demonstrate that the strain-induced metallic band structure in **b** completely reverts to the initial insulating state upon the release of the applied strain in **c**.

Referee comment: *2. According to the DFT calculations, the CDW gap within EB ranges of 0.7–0.9 eV increases when the system changes from AL to L stacking (Figs. 3e and 3f). However, experimentally, the spectral intensity within the CDW gap increases under strain, and the energy position of the gap edges remains fixed (Fig. 1k). Could the authors clarify this apparent discrepancy?*

Our response: We thank the referee for this careful observation. We have thoroughly re-examined our data and found that this discrepancy originates from matrix-element effects in the ARPES measurements. While we plotted the ARPES spectra at k_x = +0.65 Å^{-1} in

previous Fig. 1k, the analysis of the spectra at the symmetrically identical momentum k_x = −0.65 Å^{-1} (shown in Fig. R8c) reveals that the CDW gap for the AL stacking is indeed smaller than that for the L stacking (as indicated by black dashed lines). This is consistent with the DFT calculations.

This observation indicates that, among the three subbands expected for the L stacking (indicated by green, orange, and white triangles in Fig. R8d), the photoemission intensity of the subband marked by the orange triangle is strongly suppressed at k_x = +0.65 Å^{-1} due to matrix-element effects, which led to an overestimation of the CDW gap size in our initial plot. It is noted that we have incorrectly defined the gap size as the energy difference between green and white arrows. On the other hand, at k_x = −0.65 Å^{-1}, the observation of the subbands indicated by the green and orange triangles enables a correct estimation of the gap size, (note that the subband indicated by white triangle is weak).

To discuss the intrinsic band structure and resolve this discrepancy, we have replaced the original Fig. 1h,i,k with Fig. R8a-c in the revised manuscript. In addition, we have

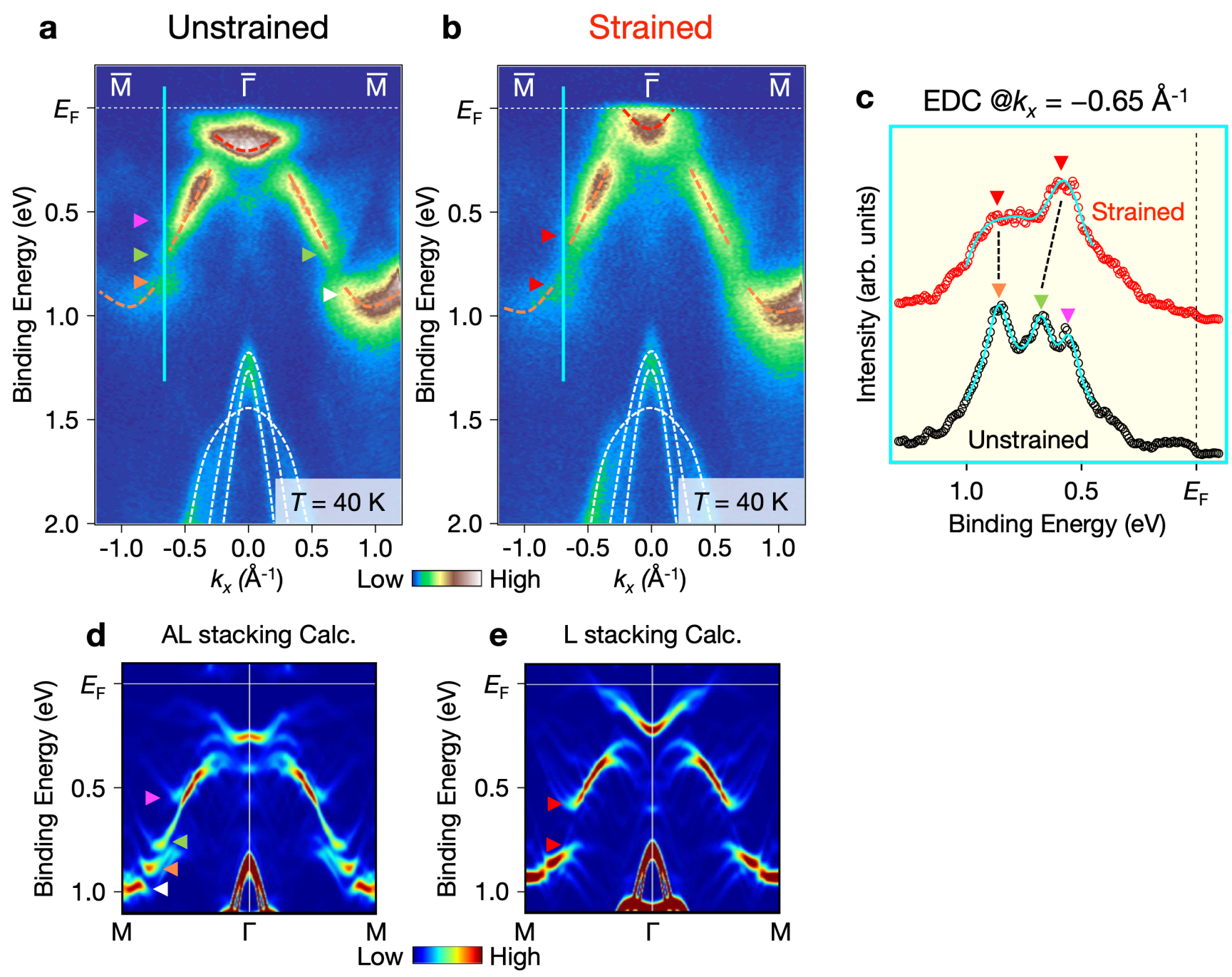

Fig. R8 | Re-examination of the CDW gap size. **a**, **b** ARPES-intensity plot measured along the $\overline{\Gamma M}$ cut with and without strain, respectively. **c** Comparison of EDCs between the unstrained and strained samples at k_x = −0.65 Å^{-1} shown by cyan lines in **a** and **b**, together with the results of numerical fittings (solid curves). **d**, **e** Calculated electronic structures for L and AL stacking configurations respectively, along the ΓM line.

revised the related sentence as "the CDW gap magnitude appears to increase (Fig. 1k), as the energy separation of the gap edges is larger." in the main text (p. 7, lines 137–138).

Referee comment: *3. Since the application of tensile strain in this context is somehow technically innovative, more details should be provided regarding the characterization of strain. For example, the use of finite-element simulations and strain-gauges on page 6.*

Our response: We thank the referee for this valuable encouragement. The finite-element simulations were performed using SOLIDWORKS Simulation. For the metal plate made of CuBe, a Young's modulus of 115 GPa and a Poisson's ratio of 0.3 were implemented. Figure R9a shows the simulated spatial distribution of the axial strain along x (ε_{xx}) on the TaS_2 sample with realistic dimensions of 1×1×0.05 mm^3 glued on a CuBe plate. The absence of a strain gradient along the y axis supports the uniaxial nature of the applied strain. On the other hand, along the x axis, the strain macroscopically varies from ~1% at the center of the sample to ~0.2% near the edges, corresponding to a gradient of 1.6%/mm. Within the ~12-μm width of the beam spot in our micro-ARPES system, this gradient corresponds to a strain variation of only ~0.02%, which is two orders of

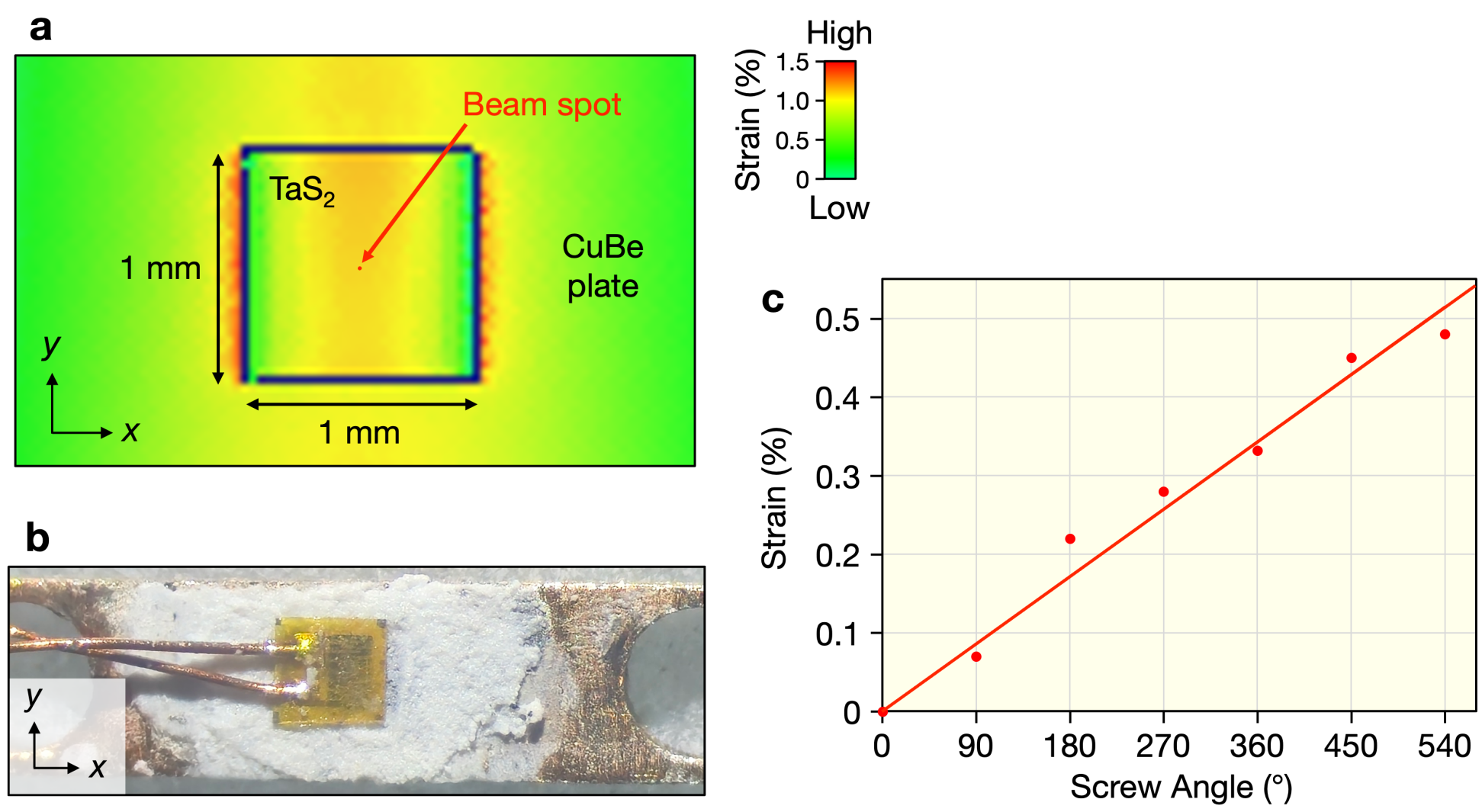

Fig. R9 | Characterization of applied strain. **a** Spatial distribution of tensile strain (ε_{xx}) assuming a 1×1×0.05 mm^3 sample glued on CuBe plate, simulated by the finite element method. **b** Optical microscope image of the strain gauge on the sample plate used to measure the actual magnitude of the strain. **c** Plot of strain gauge readings against rotation angle of the pressure adjustment screw (red dots) with a linear fit (red line).

magnitude smaller than the applied maximum strain. This negligible gradient ensures that the strain can be considered uniform within the probed region.

Regarding the characterization of the actual magnitude of the applied strain, we used commercial strain gauges (KYOWA KFRS-02-120-C1-13) at room temperature (see Fig. R9b for the measurement configuration). As shown in Fig. R9c, upon rotating the screw of the device, the strain measured by the gauge increases, reaching a maximum of 0.5%. The smaller magnitude of the measured strain than the maximum simulated value (approximately 1%) is attributed to the spatial averaging effect; as mentioned above, there is a macroscopic gradient of the strain along x, and the dimension of the strain gauge used in this study is 0.8 mm in width, so that the measured results represent a spatial average of the strain distribution. Therefore, the 0.5% sets the minimum strain magnitude and the actual maximum strain must be larger.

We have described these points in the Methods section (pp. 14–15, lines 288–293) and Supplementary note 1.

<u>Referee comment</u>: *4. The DFT calculation details are essential, as they lay the foundation for interpreting changes in stacking order changes and band renormalization. These should be explicitly described in the Methods section and cannot be omitted.*

<u>Our response</u>: Following the referee's suggestion, we have added the following sentences in the Methods section:

First-principles calculations were based on the density functional theory (DFT) within the generalized gradient approximation (GGA) [17]. The cut-off energy was set at 260 eV, and the k-point mesh was 4×4×8 for the Brillouin zone of the √13×√13×1 cell. All atoms were relaxed, and the calculated equilibrium lattice constants (a = 3.35 Å and c = 5.79 Å) reasonably agree with the experimental values (a = 3.36 Å and c = 5.92 Å).

<u>Referee comment</u>: *5. On page 6, the term "confirming high single crystallinity of our sample" may be misleading. ARPES can reveal coherent band dispersion and a weak inelastic background, which support good crystallinity, but cannot definitively confirm single crystallinity from the observation of "several highly dispersive bands".*

<u>Our response</u>: We thank the referee for careful reading. We have replaced "high single crystallinity" with "good crystallinity" in the revised manuscript.

Finally, we thank all the referees again for their thoughtful and constructive comments. We believe that the manuscript has been substantially improved through the revision, and the key findings and their significance are now more clearly presented. We hope that the revised manuscript will be suitable for publication in Nature Communications.

Sincerely,

Kosuke Nakayama and Takafumi Sato, on behalf of all authors
Department of Physics and WPI-AIMR,
Tohoku University

Response to referees (second revision):

We sincerely thank all the referees for their continued evaluation of our manuscript and for their constructive comments. Following the editor's recommendation and the helpful suggestions of the First Referee, we carried out a more quantitative analysis of the tensile strain at the surface of a representative TaS_2 crystal using laser microscopy. Within a simple Poisson-ratio approximation, the interlayer compression is estimated to reach ~0.4%, corresponding to the regime in which the AL and L stacking configurations become nearly degenerate. This analysis strengthens the physical basis of our interpretation and clarifies the distinction between the present work and Ref. 32.

Below we provide a point-by-point response to each comment. The referees' comments are shown in blue italics.

To the comments from the First Referee:

Referee comment: *The revised manuscript argues that the applied uniaxial tensile strain induces a surface-localized stacking rearrangement from an AL-like dimerized stacking toward an L-like, non-dimerized configuration. This interpretation is plausible, especially in view of the small energy difference between AL and L stackings discussed by Lee et al. PRL 122, 106404 (2019) and Nicholson et al. PRB 109, 035167 (2024). I would suggest the publication of the work. However, the manuscript would be strengthened to make a more quantitative connection between the externally applied bending strain and the proposed near-surface stacking and electronic reconstruction.*

Our response: We sincerely thank the referee for recommending publication. In response to this helpful suggestion, we have strengthened the quantitative connection between the applied strain and the proposed near-surface reconstruction, as detailed below.

Referee comment: *1. The authors discuss the possibility that the in-plane tensile strain reduces the out-of-plane interlayer distance through a Poisson response, and that the bending geometry may also induce a surface-enhanced shear and mild slip component. Could the authors estimate the actual strain tensor in the TaS_2 crystal near the ARPES-probed surface, in particular the out-of-plane strain ε_{zz} and the relevant shear component, for the nominal applied ε_{xx} ~ 1%? This would be useful because previous works (Lee et al. & Nicholson et al.) suggested that AL and L stackings can become nearly degenerate for a reduction of the interlayer spacing of order ~0.3%. If the effective Poisson response leads to ε_{zz} in this range, the proposed AL-to-L tendency would be quantitatively supported. If not, it would be important to clarify whether the dominant driving mechanism is instead the surface-localized shear/slip relaxation.*

Our response: We thank the referee for this important suggestion. While a quantitative estimation of the shear/slip component in the present bending geometry is difficult from our measurements, we were able to estimate a representative value of the near-surface out-of-plane strain ε_{zz}. Its magnitude is approximately 0.4%, comparable to the ~0.3% reduction in interlayer

spacing at which the AL and L stackings are predicted to become nearly degenerate. This provides quantitative support for a tendency toward an L-like stacking rearrangement. The details of this estimate are described below.

We estimated the near-surface strain in two steps: (i) an *ex situ* laser-microscopy measurement of the local in-plane strain ε_{xx} and (ii) an estimate of the corresponding out-of-plane strain ε_{zz}. Because the TaS_2 sample used for the laser microscopy was different from the one used for ARPES but was mounted on the same strain device [Fig. RR1(a)], the resulting values should be regarded as representative estimates of the strain scale relevant to the ARPES observation.

Regarding (i), we acquired laser-microscope images of the TaS_2 crystal at room temperature in air using a KEYENCE VK-X1100 laser scanning confocal microscope before and after applying uniaxial strain along the x axis [Figs. RR1(b) and RR1(c), respectively]. Bright and dark features associated with surface morphology were visible in both images and served as spatial markers. We extracted line profiles along the red line near the center of the crystal, at a position geometrically corresponding to the region nominally probed by micro-ARPES, and observed an increase in the lateral distance between matched features [compare the black and red dotted lines in Fig. RR1(d)]. This analysis reveals a local in-plane tensile strain $\varepsilon_{xx} \approx 1.5\%$, in reasonable agreement with the ~1.3% value obtained from our finite-element simulations. We therefore used 1.5% for the following estimate of ε_{zz}.

Regarding (ii), using a Poisson ratio $\nu = 0.26$ previously adopted for TaS_2 [Chernolevska *et al.*, Sci. Rep. **15**, 36052 (2025)], the representative ε_{xx} value ≈1.5% gives $\varepsilon_{zz} = -\nu\, \varepsilon_{xx} \approx -0.39\%$. This value is comparable to the 0.3% reduction in interlayer spacing at which prior calculations place the AL and L stackings near degeneracy (Lee *et al.*; Nicholson *et al.*).

The in-plane tension and resulting out-of-plane compression may therefore reduce the energy cost of an L-like stacking rearrangement, while surface-enhanced shear/slip may further assist such a rearrangement. We believe that these additional results and analyses provide an important new physical insight beyond that available in Ref. 32.

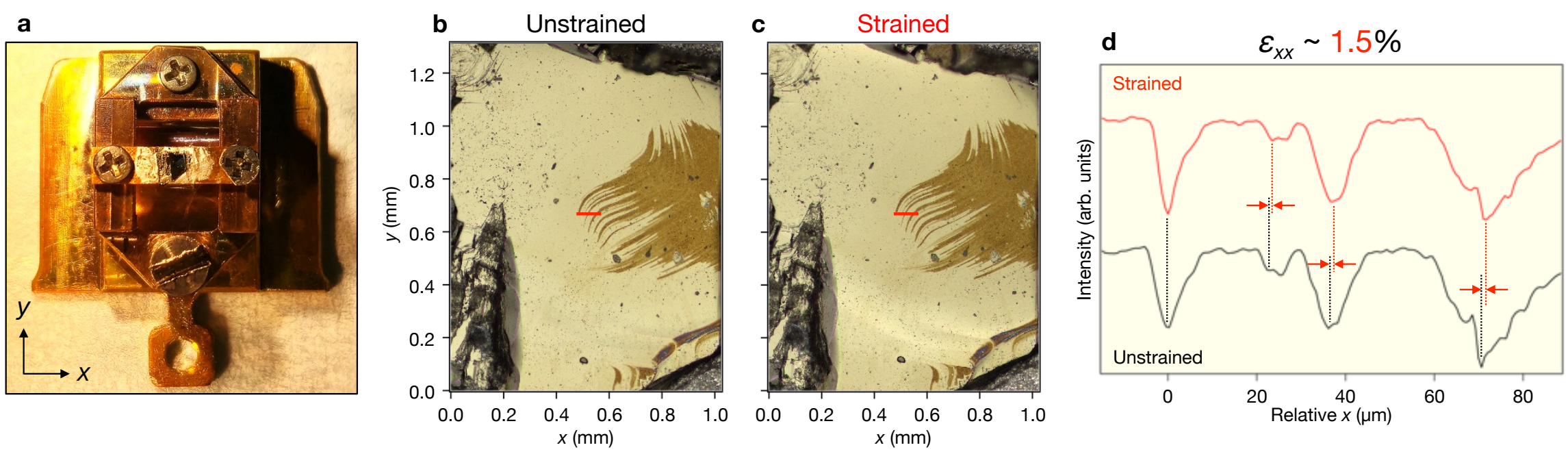

Fig. RR1 | Calibration of the surface in-plane strain on a representative sample by laser microscopy. a Photograph of the strain device. **b, c** Laser-microscope images of a TaS_2 surface for the unstrained and strained states, respectively. **d** Intensity line profiles for the unstrained (black) and strained (red) states along the red line in **b**,**c**, from which the local in-plane strain ε_{xx} is evaluated.

We have added the laser-microscopy strain measurement as new Supplementary Fig. S2 and have described these estimates in the revised manuscript (p. 6, lines 116–119; p. 10, line 211 – p. 11, line 213) and Supplementary note 1.

Referee comment: *2. The newly added XRD data show that AL-associated satellite peaks remain visible under strain, indicating that the bulk remains at least partially AL-like. If the ARPES metallicity originates from a surface-localized L-like configuration, it would be helpful to estimate how many topmost layers are expected to undergo such a reconstruction, and whether such a surface-confined region is sufficient to dominate the ARPES signal given the photoelectron escape depth.*

Our response: We thank the referee for this important suggestion. The photoelectron mean free path λ in our ARPES measurements at $h\nu = 98$ eV is approximately 5 Å [M. P. Seah and W. A. Dench, Surf. Interface Anal. **1**, 2 (1979)], comparable to the interlayer spacing of TaS_2 (c = 5.92 Å). Assuming that the n-th Ta layer is located at $z_n = (n - 1/2)c$ below the surface and that the photoemission intensity is attenuated as $\exp(-z_n/\lambda)$, we obtain attenuation factors of 0.553, 0.169, 0.052, and 0.016 for the first four Ta layers, corresponding to normalized contributions of 69.4%, 21.2%, 6.5%, and 2.0%, respectively, to the total ARPES signal. Thus, the topmost two Ta layers account for approximately 91% of the total ARPES signal. The absence of a clearly discernible insulating component in the strained ARPES spectra therefore suggests that the L-like reconstruction extends over at least the topmost two TaS_2 layers.

Such a surface-confined reconstruction is consistent with our XRD results, which indicate that the AL stacking remains dominant in the bulk. Because the XRD probing depth is orders of magnitude larger than the thickness of a few TaS_2 layers, a thin reconstructed surface region would make a negligible contribution to the total diffraction intensity. The XRD and ARPES results are therefore consistent with an L-like reconstruction in the near-surface layers that dominate the ARPES signal.

We have described these points in the revised manuscript (p. 11, lines 217–219) and Supplementary note 6.

Referee comment: *3. The proposed bending-induced shear/slip mechanism may also produce an intermediate structure, in which the A-stacked dimer is laterally displaced only partially. Could the authors clarify whether the expected shear/slip is large enough to reduce A-stacking dimer hopping to an L-like value and fully close the dimerization gap, or whether the observed metallic ARPES spectrum could instead arise from a partially slipped AL dimer with substantially weakened interlayer hybridization?*

Our response: We thank the referee for this comment. The present measurements alone do not allow us to exclude an intermediate partially slipped configuration. Resolving the detailed atomic stacking would require direct structural characterization, for example by cross-sectional transmission electron microscopy, which is beyond the scope of the present work. As described in our response to the referee's comment 1, the representative ε_{zz} value is $\approx -0.4\%$, a scale at

which the energy difference between the AL and L stackings is expected to be very small. Under such conditions, even a relatively small shear perturbation induced by bending could facilitate a rearrangement toward an L-like stacking configuration. We have described this point in Supplementary note 5. We also slightly soften the statement "a transition in the interlayer stacking from the dimerized AL configuration to the non-dimerized L configuration" to "a rearrangement of the interlayer stacking from the dimerized AL configuration toward an L-like configuration" in the revised manuscript (p. 10, lines 204–205).

To the comments from the Second Referee:

Referee comment: *This is my second round of review for this manuscript. The authors argue that they achieved a larger nominal strain than Ref. 32 (PRB 109, 035167 2024), but this is a quantitative rather than a qualitative difference and the observed evolution of the band structure—specifically the reduction of the electronic gap—follows the same trend already reported. The fact that the gap becomes smaller and eventually closes under larger strain is expected. Moreover, the ARPES data in the present manuscript are of poorer quality than those in Ref. 32 (band dispersion near the Fermi level is clearly observed with 6.2 eV laser ARPES in Ref. 32), making it difficult to confidently identify a clear insulator-to-metal transition. Furthermore, no dramatic change in band dispersion is observed across the transition.*

Additionally, Figure 3 of the present manuscript bears a strong resemblance to Figure 3 of Ref. 32, both in terms of the stacking-dependent band structure and the overall conclusions.

Therefore, the claimed advances are incremental at best, and the manuscript lacks the conceptual or technical novelty required for publication in Nature Communications.

Our response: We thank the referee for taking the time to evaluate the revised manuscript. As the referee pointed out, the reduction of the band gap by applying strain was reported in Ref. 32. However, we respectfully disagree that the present work represents only a quantitative increase in the applied strain. The larger applied strain enables access to a qualitatively different electronic state: namely the metallic regime, whereas Ref. 32 remained in the gapped regime. Therefore, the difference is not merely quantitative but also qualitative.

Beyond this qualitative difference, our work advances the quantitative understanding of the microscopic origin of the strain-induced metallic state. In response to the referees' comments, we added analyses that were not available in Ref. 32. Specifically, we (i) performed a laser-microscopy measurement of the local surface strain, (ii) estimated the corresponding out-of-plane compression using the Poisson effect, (iii) showed that the estimated compression reaches the theoretically predicted near-degenerate regime between the AL and L stackings, and (iv) quantitatively reconciled the apparently different sensitivities of ARPES and XRD by evaluating the photoelectron escape depth and the corresponding reconstruction depth. Access to the metallic regime also enabled the observation of a strongly renormalized half-filled band, which was not available in Ref. 32. Although the stacking-dependent calculations naturally resemble those of Ref. 32 because both studies compare the same representative AL and L

configurations, the present work extends the microscopic interpretation of the strain-induced metallic phase through these new quantitative and surface-sensitive analyses, which were not available in Ref. 32.

To better emphasize these conceptual and quantitative distinctions from Ref. 32, we have revised the manuscript to incorporate the new analyses described above (see our responses to Comments 1 and 2 of the First Referee).

To the comments from the Third Referee:

Referee comment: *I thank the authors for their careful revisions and detailed responses to my previous comments. All of my concerns have been satisfactorily addressed. In particular, the additional experiments have strengthened the paper. I believe the manuscript is now suitable for publication in Nature Communications and have no further requests for revision.*

Our response: We sincerely thank the referee for the positive evaluation of our revised manuscript and for recommending its publication in Nature Communications.

We thank all the referees and the editor again for their thoughtful and constructive comments, which have allowed us to place our interpretation on a firmer quantitative footing. We hope that the revised manuscript is now suitable for publication in Nature Communications.

Response to reviewers:

We sincerely thank all the reviewers for their evaluation of our manuscript. We are delighted to learn of the positive decision regarding our manuscript. Below we describe our response to the final comments from the Reviewer #2, which are shown in blue italics.

Reviewer comment: *I have read the authors' reply and the revised manuscript. However, my primary concern regarding the novelty of the work remains unaddressed. The present study relies on an identical experimental setup and the sample system as those detailed in Ref. 32 (Supplementary Methods). The only distinguishable difference is the authors' assertion that applying higher strain drives the system into a "metallic" state, which they appear to acknowledge. This metallicity is supported solely by a shift in the spectral edge, a phenomenon that was already observed, albeit to a lesser extent, in Ref. 32.*

Our response: As this point is essentially the same as that raised in the previous round, we refer to our previous response and the corresponding revisions to the manuscript. Briefly, while Ref. 32 reported a strain-induced reduction of the gap, the system remained gapped. In contrast, the present study reaches the metallic regime, as supported not only by the emergence of a Fermi edge but also by the observation of a Fermi-surface pocket. The key point is that this difference is not merely quantitative, as suggested by the reviewer, but qualitative, since the insulating and metallic states represent distinct electronic regimes. Access to this metallic regime further enables us to characterize the half-filled quasiparticle band and its mass renormalization, which was not possible in Ref. 32. We therefore believe that the present work accesses a qualitatively distinct electronic regime beyond the strain range explored in Ref. 32.

Reviewer comment: *The XRD results directly contradict the ARPES-based conclusion. The authors attempt to resolve this by invoking an L-like reconstruction in the near-surface layers, while asserting that AL stacking persists in the bulk. This interpretation, however, rests on conjecture. In fact, a more straightforward explanation is that the ARPES signal originates from strain-induced lattice constant change, stacking faults and/or disorder, which would naturally produce the observed spectral shifts.*

Our response: We agree with the reviewer that the present XRD and ARPES measurements do not uniquely establish an ideal long-range-ordered L stacking at the surface. We already acknowledged this limitation in our response to Reviewer #1 in the previous round and therefore use the term "L-like" to describe a near-surface stacking environment in which the AL-type interlayer dimerization is substantially weakened. In this sense, stacking faults involving deviations from the A-stacked dimer configuration resemble the partial interlayer slip discussed in the manuscript and hence are compatible with our interpretation of an L-like near-surface reconstruction. We have described this point in the revised manuscript (p. 11, lines 255–257 in the main text; p. 6, lines 2–3 of the third paragraph in Supplementary Note 5).

As discussed in our previous response to Reviewer #1, however, the stacking disorder associated with the random choice among the three symmetry-equivalent L sites does not

necessarily disrupt the A-stacked interlayer dimers and therefore would not, by itself, be expected to close the band gap. Also, a uniform strain-induced change in the lattice constants would not by itself close the gap as long as the AL-type interlayer dimerization is preserved, because the gap originates from the dimerization. We therefore retain the interpretation that a modification of the near-surface interlayer stacking/dimerization is required to account for the metallic state. Future depth-resolved structural characterization will be important for determining the detailed near-surface stacking configuration and clarifying the microscopic nature of the strain-induced reconstruction.
